# Risk factors for delirium after on-pump cardiac surgery: a systematic review

**DOI:** 10.1186/s13054-015-1060-0

**Published:** 2015-09-23

**Authors:** Alex NC Gosselt, Arjen JC Slooter, Pascal RQ Boere, Irene J Zaal

**Affiliations:** Department of Intensive Care Medicine, University Medical Center Utrecht, Heidelberglaan 100, 3584 CX Utrecht, The Netherlands

## Abstract

**Introduction:**

As evidence-based effective treatment protocols for delirium after cardiac surgery are lacking, efforts should be made to identify risk factors for preventive interventions. Moreover, knowledge of these risk factors could increase validity of etiological studies in which adjustments need to be made for confounding variables. This review aims to systematically identify risk factors for delirium after cardiac surgery and to grade the evidence supporting these associations.

**Method:**

A prior registered systematic review was performed using EMBASE, CINAHL, MEDLINE and Cochrane from 1990 till January 2015 (http://www.crd.york.ac.uk/PROSPERO/display_record.asp?ID=CRD42014007371). All studies evaluating patients for delirium after cardiac surgery with cardiopulmonary bypass (CPB) using either randomization or multivariable data analyses were included. Data was extracted and quality was scored in duplicate. Heterogeneity impaired pooling of the data; instead a semi-quantitative approach was used in which the strength of the evidence was graded based on the number of investigations, the quality of studies, and the consistency of the association reported across studies.

**Results:**

In total 1462 unique references were screened and 34 were included in this review, of which 16 (47 %) were graded as high quality. A strong level of evidence for an association with the occurrence of postoperative delirium was found for age, previous psychiatric conditions, cerebrovascular disease, pre-existent cognitive impairment, type of surgery, peri-operative blood product transfusion, administration of risperidone, postoperative atrial fibrillation and mechanical ventilation time. Postoperative oxygen saturation and renal insufficiency were supported by a moderate level of evidence, and there is no evidence that gender, education, CPB duration, pre-existent cardiac disease or heart failure are risk factors.

**Conclusion:**

Of many potential risk factors for delirium after cardiac surgery, for only 11 there is a strong or moderate level of evidence. These risk factors should be taken in consideration when designing future delirium prevention strategies trials or when controlling for confounding in future etiological studies.

**Electronic supplementary material:**

The online version of this article (doi:10.1186/s13054-015-1060-0) contains supplementary material, which is available to authorized users.

## Introduction

Postoperative delirium is a frequent condition after cardiac surgery, with reported frequency between 3 % [1] and 31 % [2–7]. Postoperative delirium is associated with short-term and long-term morbidity and mortality [8–11], and consequent raised health care costs [12, 13].

To date, well-established treatment options for postoperative delirium are lacking [14–17]. This emphasizes the importance of applying prophylactic strategies to decrease the burden of delirium after cardiac surgery. Knowledge as to whether there is supporting or conflicting evidence for an association between different predisposing and precipitating factors and delirium, will provide guidance to clinicians on opportunities for prevention. Moreover, knowledge about risk factors will inform researchers about the key variables that should be incorporated in future multivariable models for the analysis of postoperative delirium.

Earlier systematic reviews on this topic have limited validity as they included heterogeneous study populations within limited search strings and time-windows and because they applied quantitative measures to identify strong risk factors without taking the quality of the studies into account [18, 19]. The aim of this study was to systematically review the literature on potential risk factors for delirium after cardiac surgery in which cardiopulmonary bypass (CPB) was performed.

## Methods

### Design

The review was performed in accordance with the Preferred Reporting Items for Systematic Reviews and Meta-Analysis statement recommendations for conducting a systematic review [20]. Both the study protocol [58] and the full search strategy [58] were registered before initiation of the search.

### Eligible studies

Five databases were searched (CINAHL, EMBASE, MEDLINE, the Cochrane Central Register for Controlled Trials, and the Cochrane Database of Systematic Reviews) for relevant articles or abstracts published from January 1990 through January 2015. With an online registered comprehensive search strategy using separately formulated strings for the domain (cardiac surgery patients) and the outcome (delirium) we searched for eligible studies. We deliberately left out the determinant (risk factors) to lower the possibility of missing relevant articles. We chose 1990 as the initial search year because this was the year that a well-defined screening instrument for delirium based on the *Diagnostic and Statistical Manual of Mental Disorders III*-revised (DSM III-R) definition was published [21].

### Study selection

All abstracts and titles were screened in duplicate for potentially relevant studies, which were considered in full-text form by two authors (ANCG and PRQB). Whenever in doubt, studies were discussed with a third author (IJZ) before inclusion. We reviewed personal files, reference lists of review articles, and reference lists of eligible studies for additional investigations to identify additional relevant publications that were missed during the computerized search. We contacted the corresponding author if no full text version of the article was available to us or to inquire for missing data. Corresponding authors who failed to respond after the first contact were contacted one additional time over a 6-week period.

Cohort studies or randomized controlled trials were included that evaluated adults (age ≥18 years) undergoing cardiac surgery using CPB, where at least one potential risk factor for delirium was considered. The risk factor had to be present before delirium onset, and all patients had to have been evaluated for delirium at least once daily using a validated instrument. We excluded studies that also evaluated patients undergoing cardiac surgery without CPB or solely aortic surgery because of possible differences in the pathobiology of delirium. Further, cohort studies that failed to evaluate risk factors using a multivariable approach were excluded given the multitude of possible confounding variables. Articles published in a language other than English, Dutch or German were excluded.

### Data extraction

All data on the risk factor selection process and final association was independently extracted by two authors (ANCG and PRQB). Variables with an uncertain cause − effect relationship were excluded from the final tables. Discrepancies were resolved through discussion with a third author (IJZ) when necessary. In the case of separate studies using the same study cohort for the analysis, significant variables in both studies were counted only once.

### Assessment of the risk of bias

Two authors (ANCG and PRQB) independently assessed the risk of bias in each included study by using adapted versions of the Scottish Intercollegiate Guidelines Network (SIGN) checklist for controlled trials and cohort studies [22].

The SIGN RCT checklist considered randomization strategy, treatment allocation concealment, blinding, success of randomization, use of intention-to-treat principles and the completeness of the reported outcome data (see Additional file 1A). The SIGN checklist for cohort studies was modified a priori by consensus. The checklist for cohort studies considered selection bias, performance bias, loss to follow up resulting in attrition bias, detection bias and statistical analysis. In order to be included in this study, a validated assessment for delirium had to be done either by use of the DSM III-R criteria [23] (or a newer version), or by a delirium screening tool which had to be validated for the specific population e.g., ventilated/ICU patients (Additional file 1B).

If a checklist criterion was met, one point was assigned, studies that failed to meet a criterion or provided insufficient information, resulting in a ‘cannot state’, and received no points. The maximum attainable score was 8 points for cohort studies and 9 points for randomized controlled trials (RCTs). ‘Not applicable’ items were counted as one point as it was deemed not to be a consequence of insufficient study quality. Items deemed ‘not applicable’ did not illustrate methodological shortcomings and were counted as a point.

If a secondary multivariable analysis was performed within an RCT to identify independent risk factors other than the main factor under investigation, this analysis was separately assessed for bias using the cohort checklist. Any disagreement during the quality scoring process was resolved by discussion with a third author (IJZ). A priori, cohort studies and RCTs were deemed high quality (HQ) when the score was ≥7 and ≥8 points, respectively, acceptable quality (AQ) when the score was 5–6, and 6–7, respectively, and unacceptable quality when the score was ≤4, and ≤5 points, respectively.

### Data synthesis

Strength of evidence supporting an association between a risk factor and delirium was summarized using a semiquantitative approach. Variables that were analyzed using a multivariable approach, in at least two studies and that showed either a statistical significant association or an odds ratio ≤0.5 or ≥1.5 were included in the best evidence synthesis. Due to the fair amount of studies with small sample sizes we did not solely want to depend on statistical significance as this is highly dependent on sample size and other included variables. If an HQ study described a variable to be initially included in a stepwise selection process but was not presented in the final model it was categorized as having no association with delirium in a multivariable approach.

The available evidence for potential risk factors for postoperative delirium after cardiac surgery was quantitatively evaluated using three criteria: 1) the number of studies evaluating the variable, 2) the scored quality of each study evaluating the variable, and 3) the consistency of the association between the variable and risk of delirium. An association was deemed consistent when ≥75 % of the studies evaluating the variable reported the same direction of association. Variables found to have no association using a multivariable approach were also taken into account.

A variable evaluated at different time-points (e.g., perioperative or intraoperative blood product transfusion), or a variable for which slightly different definitions were used, was considered similar and therefore grouped. Details describing these differences are specified in Additional file 2.

We graded the strength of evidence for each association as follows: strong when the association was consistent in ≥2 HQ studies; moderate when consistent in 1 HQ study and ≥1 average quality (AQ) study or ≥3 AQ/LQ studies; inconclusive when the observed association was not consistent or was evaluated in 1 HQ, <3 AQ studies or solely in LQ studies. We concluded no evidence for an association if no significant association was found in HQ multivariable analysis or at least 3 HQ studies found no association in univariable analysis (Table 1).

**Table 1:** Level of evidence for identification of risk factors for postoperative delirium

Level of evidence	Criteria
Strong	Consistent findings (≥75 %) in ≥ 2 high-quality articles
Moderate	Consistent findings (≥75 %) in 1 high-quality article and ≥1 acceptable-quality article or ≥3 acceptable or low-quality articles.
Inconclusive	Inconsistent findings irrespective of study quality or 1 high-quality article or <3 acceptable/low-quality articles
No association	No association in multivariable analysis in high-quality articles and ≥3 high-quality articles with no association in high-quality articles

## Results

### Study identification

**Fig. 1:**
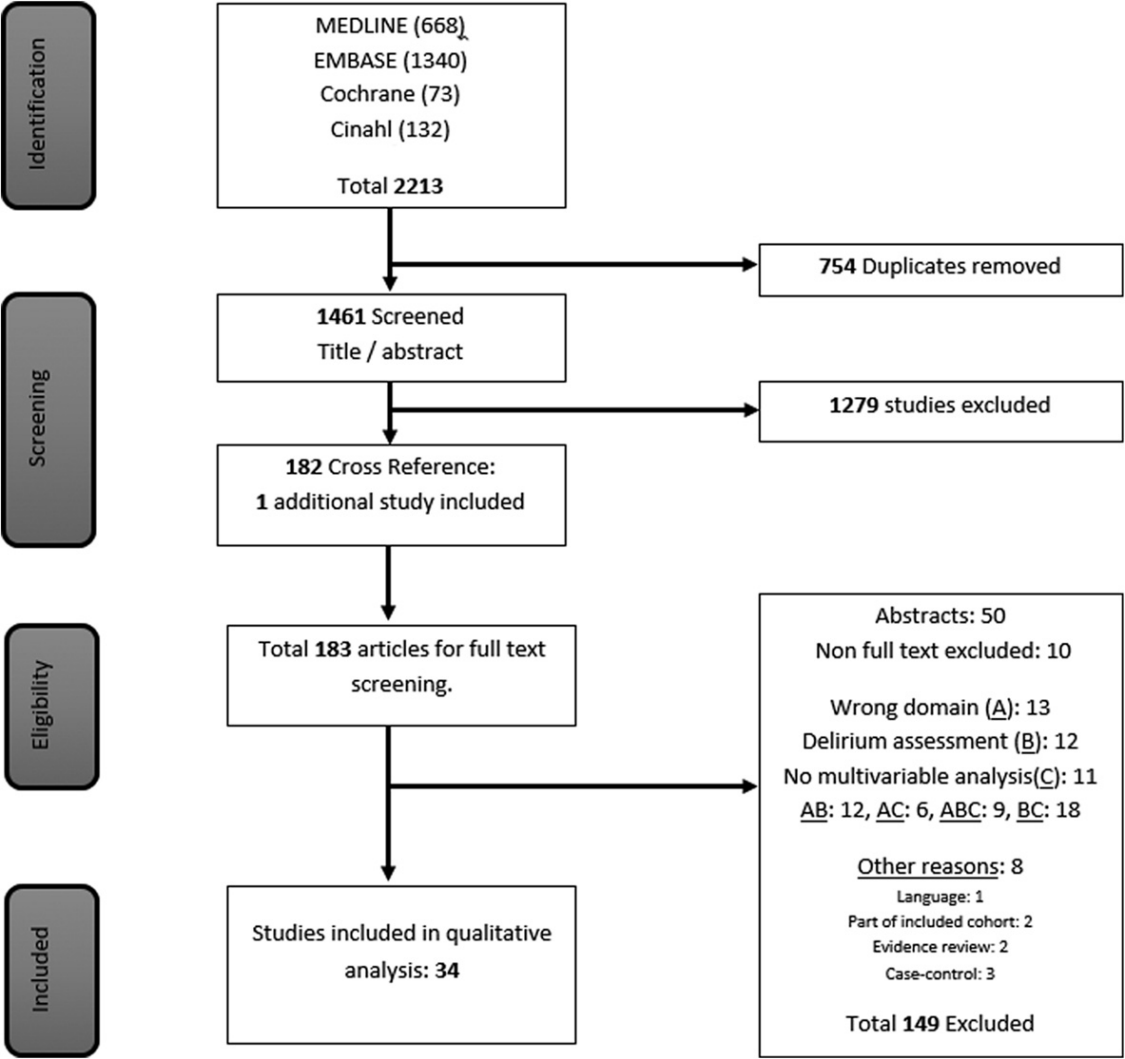
Flowchart of study selection. Of 2,213 references, 34 studies (26 cohort, 8 randomized controlled trials) were eligible for inclusion

The search yielded 1,462 unique references of which a total of 1,279 references were excluded based on a review of the title and abstract, leaving 183 references for full-text review. Of these, 50 solely represented an abstract for a conference presentation, and 10 articles for which the full text was unavailable, had no contact information or lacked a response, and were consequently excluded. Of the remaining 123 articles, 13 did not refer to an appropriate domain, 12 did not use delirium as an outcome, or used a subjective or non-validated delirium assessment tool, 11 used no multivariable approach or randomized design, 45 had a multitude of these reasons, and 8 were excluded for other reasons. Therefore, a total of 34 studies were included for systematic review (see Fig. 1 for flowchart of study selection, and Additional file 3).

The 34 studies included consisted of 24 cohorts, 2 before/after observational studies and 8 RCTs; of which 4 also used a multivariable cohort approach to identify other risk factors. Three cohort studies were based on the same study population but presented models on different risk factors [24, 25, 57].

### Study characteristics

Sample size in the 34 included studies varied from 36 to 4,079 patients, with a variation in delirium incidence of 2.9 − 54.9 %. Several studies limited their study population to patients undergoing coronary artery bypass graft (CABG) and/or valve surgery only; others refrained from defining cardiac surgery or included thoracic aortic surgery. For 28 studies use of CPB was confirmed in all patients, the use of CPB in the remaining 6 studies was uncertain, however, as off-pump surgery was not mentioned either, we retained those for our analysis. In most studies only patients undergoing elective surgery were included; four studies did not mention surgical urgency. The confusion assessment method (CAM) and/or the adjusted version for the ICU (CAM-ICU) were used most extensively (n = 19). In most studies researchers or trained research nurses assessed delirium, and the assessor was not described in one study [4]. One RCT included patients with sub-syndromal delirium, aiming to prevent progression to full-blown delirium [26]. Follow-up time varied with six studies only assessing patients during their ICU stay, six started at postoperative day 2 (mostly to exclude the residual effect of anesthetic drugs) and two did not fully report the duration of follow up (see Additional files 4 and 5).

### Methodological quality

The results of the quality analysis according to the SIGN checklists are presented in Additional files 6 and 7. Of 30 cohort studies, 13 (43.3 %) were scored as HQ studies, 13 (43.3 %) as AQ and 4 (13.3 %) as LQ studies. Of the eight RCTs, five were graded as HQ, two as AQ and one as LQ. Several studies did not report delirium assessment preoperatively, indicating the reported frequencies of delirium could refer to the prevalence rather than the incidence. Two studies included urgent surgery, but preoperative mental status assessment was not performed; these studies were deemed to have high risk of performance bias [27, 28].

Several studies had detection bias; 16 due to limited follow up, e.g., only ICU assessments or exclusion of postoperative day 1, or both day-2 and day-3 assessments. In another five studies [29–33], delirium detection was likely influenced by including ICU/ventilated patients for which the assessment tool (CAM/DOS) was not validated. One study used retrospective chart review using DSM-IV criteria for diagnosis of delirium [28]. Blinding of exposure was deemed not applicable in most cohort studies evaluating basic risk factors, e.g., age. In before − after studies [27, 31], and those studying baseline cognitive/executive functioning [30, 32, 34–36], failure to address this issue resulted in possible bias. Five studies investigated the association between delirium and a risk factor that was not properly defined [4, 28, 37–39] or validated [31]. Most studies had fairly appropriate statistical models, although one was graded lower quality as the final multivariable model included only length of stay and mechanical ventilation time [4], another failed to include the before − after intervention in the statistical model [27].

The eight RCTs had a median quality of 9 (range 5–9). One study used CAM in ICU patients and received no point for outcome measurement [3]. Two studies compared dexmedetomidine with morphine or propofol. Due to open-label use of other sedatives and analgesics, the treatment groups were not similar, making conclusions about the intervention cumbersome [40, 41]. The investigators were not blinded to the intervention in one study [41].

### Level of evidence for identification as a risk factor

The results of the best evidence analysis are presented in Table 2. Point estimates of the individual statistically significant variables or those with an adjusted estimate ≤0.5 or ≥1.5 are provided in Additional file 2.

**Table 2:** Best-evidence synthesis of variables associated with the occurrence of delirium reported more than once in multivariable or more than four times in univariable analysis

Variables	Multivariable analysis /randomized				Univariable analysis	Level of evidence
	HQ and positive association	positive association	negative association	no association	HQ and no association	
Predisposing variables						
Patient characteristics						
Age	[1, 45–50]	[27, 31, 37, 41, 51, 52]		[24, 36]	[53]	Strong
Gender				[41, 47, 49]	[1, 24, 36, 45, 48, 53, 54]	No association
Education				[36, 48]	[24]	No association
Nicotine use	[50]	[37]		[53]	[45, 49]	Inconclusive
Chronic pathology						
Cardiac disease/NYHA class				[1, 46]	[24, 36, 45, 49]	No association
Hypertension	[1]	[37]		[46, 49]	[24, 36]	Inconclusive
Peripheral vascular disease/atherosclerosis	[49]	[29]		[1, 46]	[36]	Inconclusive
Cerebrovascular disease	[47, 48]			[46]	[24, 36, 49]	Strong
Diabetes mellitus		[55]		[1, 46]	[36, 45, 49]	Inconclusive
Psychiatric impairment	[1, 24, 36]	[35, 51, 52]		[53]		Strong
Risk scores	[26]	[31]		[33, 41, 45, 48, 53]	[24, 36]	Inconclusive
Cognitive functioning	[24, 36, 48, 56]	[32, 34, 51, 57]			[53]	Strong
Preoperative diagnostics						
Peripheral oxygen saturation			[55]			Inconclusive
Cerebral oxygen saturation			[48]			Inconclusive
Lower hemoglobin		[35, 51, 57]		[1, 24, 48]		Inconclusive
Renal dysfunction	[1]	[39]		[24, 36]		Inconclusive

*APACHE* acute physiology and chronic health evaluation, *ACC* aortic cross-clamping, *ASA* American Society of Anaesthesiologists, *CPB* cardiopulmonary bypass, *HQ* high quality, *+* positive, − negative association, *NYHA* New York Heart Association

Eleven variables were found to have strong evidence for an association with postoperative delirium. Amongst the predisposing variables, strong evidence was found for age, previous psychiatric conditions, cerebrovascular disease and pre-existing cognitive impairment. For precipitating factors evidence was strong for type of surgery and perioperative blood product transfusion, and moderate for postoperative renal insufficiency and hypoxemia. Strong evidence was found for the association between postoperative delirium and prolonged mechanical ventilation, and postoperative atrial fibrillation, but cause − effect could not be determined. In two HQ studies a significant reduction was observed in the incidence of delirium in patients postoperatively treated with risperidone, resulting in strong evidence for this association. Additionally, there was evidence for no association between postoperative delirium and gender, education, previous cardiac disease or heart failure, and CPB duration.

## Discussion

In summary, in this thorough systematic analysis we found a strong or moderate level of evidence for an association between eleven different risk factors and delirium after on-pump cardiac surgery. These risk factors were age, previous psychiatric conditions, cerebrovascular disease, pre-existing cognitive impairment, type of surgery, perioperative administration of risperidone, blood product transfusion, postoperative oxygen saturation, mechanical ventilation time, atrial fibrillation and renal insufficiency. We found evidence for lack of an association with gender, education, CPB duration, pre-existing cardiac disease or heart failure and CPB duration.

Our review has many strengths. The PRISMA statement was used for the design of the study and the protocol was registered in advance [20, 42]. With a very comprehensive search strategy we searched several databases for publications describing potential risk factors, with an extensive time window. We included RCTs as well as cohort studies that used a multivariable approach for data analysis. We excluded studies with populations that may have important differences in delirium pathogenesis, e.g., the inclusion of solely ascending aortic surgery patients in whom certain procedures were applied (such as deep hypothermia, retrograde cerebral perfusion and aortic manipulation) that have influenced the risk of delirium, and that are not applied in other cardiac surgery patients [43]. Off-pump surgery has been shown to influence the risk of postoperative delirium [44]. By excluding studies with these patients we increased homogeneity. Furthermore, the process of screening, selection, data extraction and quality rating was performed in duplicate by two independent reviewers according to a well-defined transparent guideline.

Compared to previous attempts to review risk factors for delirium after cardiac surgery, we found convincing evidence for a smaller number of risk factors [18, 19]. In our review we also incorporated negative results, e.g., risk factors that were excluded from the final multivariate model in the different studies suggestive of lack of association. Failure to do so in previous reviews may have resulted in inclusion of ever-mentioned significant risk factors. The lack of consistency or reproducibility can indicate that the observed association resulted from bias, confounding or over-fitting of a statistical model [18, 19]. In this more thorough review we had to conclude that for many of these factors strong supporting evidence was lacking.

Additional risk factors found in this review such as perioperative administration of risperidone and postoperative oxygen saturation have either strong or moderate evidence for an association, in contrast to the previous reviews. This is a result of new published studies and the limited search strategy in the previous reviews; due to inclusion of risk factor in the search string and a shorter publication time window [19]. By including patients undergoing off-pump surgery the patient population in these previous reviews was more heterogeneous. As illustrated in Additional file 4, heterogeneity remains a problem when comparing studies. Differences in outcome measurement days, urgency of surgery, age limitations and adequacy of the screenings tools used led to striking differences in delirium incidence and the detected associations.

Unfortunately, this review also has some limitations. Even though we used a thorough search strategy, studies may have been missed. It is not possible to overcome publication bias. The cutoff scores to identify HQ studies may be arbitrary. Items deemed not applicable were counted as a point, for these were not representative of lack of study quality, but rather the consequence of the tested risk factors. Not including these points in these specific studies would have created different maximal obtainable quality scores per individual study and was therefore not feasible. This review attempted to select homogenous study populations consisting of patients undergoing on-pump cardiac surgery only. We excluded studies reporting either off-pump surgery or solely reporting ascending aortic surgery, however, some of the included studies only reported a small group designated ‘other cardiac surgery’. This could have resulted in inclusion of some patients undergoing surgery to the ascending aorta. A limitation of our approach to classify level of evidence for recently suggested risk factors is that these await confirmation by another independent study. We had to group some variables we regarded as similar, such as history of stroke, and history of cerebrovascular or neurological disease.

With regard to future research on delirium in cardiac surgery patients, more emphasis should be put on several methodological issues as outlined in this review. We only included cohort studies using a multivariable approach for data analysis to correct for confounding. However, none of the studies included all identified confounders in the model. The strength of a model largely depends on the variables included, so failure to include key variables results in non-comparable results that are not suitable for statistical pooling. As illustrated by the large variation in reported delirium incidence (2.9 − 54.9 %), selection bias and detection bias may remain an underestimated influence. We recommend incorporation of the aforementioned risk factors as well as the use of the most common assessment tools and a homogeneous study population when designing a new study into this subject in order to create generalizable results.

## Conclusion

This systematic review clarifies for clinicians and scientists which variables should be included in future multivariate models of etiological studies on delirium after cardiac surgery. Moreover it illustrates other important methodological aspects to take into account for future research into this subject. Most importantly it can provide guidance for clinicians on vulnerable patient and their characteristics when designing preventive strategies to decrease the burden of delirium after cardiac surgery.

## Key messages

- Eleven risk factors are associated with postoperative delirium after on-pump cardiac surgery
- These risk factors should be included when designing future postoperative delirium studies
- Future trials should focus on delirium-prone patients based on these risk factors

## Additional files

Additional file 1:
**Simplified Scottish Intercollegiate Guidelines Network (SIGN) methodology checklist used to evaluate studies.** (DOC 44 kb) Additional file 2:
**Effect estimates of variables tested in multivariable analysis.** (DOC 218 kb) Additional file 3:
**List of articles screened in full text review and the rationale for inclusion or exclusion.** (DOC 216 kb) Additional file 4:
**Summary of study characteristics.** (DOC 86 kb) Additional file 5:
**Description of the multivariable statistical model used in included cohort studies.** (DOC 120 kb) Additional file 6:
**Results of the methodological assessment of controlled trials.** (DOC 35 kb) Additional file 7:
**Results of the methodological assessment of cohort studies.** (DOC 71 kb)

## Abbreviations

AQ: average quality
CABG: coronary artery bypass graft
CAM: confusion assessment method
CPB: cardiopulmonary bypass
DSM-IV: Diagnostic and Statistical Manual of Mental Disorders
HQ: high quality
ICU: Intensive care unit
LQ: low quality
RCT: randomized controlled trial
SIGN: Scottish Intercollegiate Guidelines Network
DOS: Delirium Observation Screening
