# Supplementary material for: Risk factors for delirium after on-pump cardiac surgery: a systematic review
Source: Crit Care. 2015 Sep 23;19(1):346. doi: 10.1186/s13054-015-1060-0 (PMC4579578; doi:10.1186/s13054-015-1060-0)
Supplement: Additional file 1: — Simplified Scottish Intercollegiate Guidelines Network (SIGN) methodology checklist used to evaluate studies. (DOC 44 kb) [file 13054_2015_1060_MOESM1_ESM.doc]

**ADDITIONAL FILE 1**

Risk factors for delirium after on-pump cardiac surgery: a systematic review

A.N.C. Gosselt, MD., A.J.C. Slooter, MD., PhD., P.R.Q. Boere, MD., I.J. Zaal, MD., PhD.

| **ADDITIONAL FILE 1A**. Simplified SIGN methodology checklist of controlled trials | | | | | |
| --- | --- | --- | --- | --- | --- |
|  | **Type of bias** | | **Applicability and consideration for our review** | | |
| 1.1 |  | | *The study addresses an appropriate and clearly focused question* Pre-selection condition | |  |
| 1.2 | Random  Allocation | | *The assignment of subjects to treatment groups is randomised*  ‘YES’ if a good randomisation method is used such as computer generated off-site allocation ‘NO’ if deterministic methods such as day of the week, birth date, day of arrival at the clinic etc. | |  |
| 1.3 | Concealment | | *An adequate concealment method is used.*  ‘YES’ if centralized allocation, computerized allocation systems, or the use of coded identical containers ‘NO’ if no method of concealment is used or the method is regarded as poor, or relatively easy to subvert. | |  |
| 1.4 | Blinding | | *Subjects and investigators are kept ‘blind’ about treatment allocation.*  ‘YES’ if the blinding levels are single, double or triple blinded where possible.  ‘NO’ if the study could have been blinded, but was not. | |  |
| 1.5 | Successful  randomisation | | *The treatment and control groups are similar at the start of the trial.*  ‘YES’ if the patient groups look reasonably similar. ‘NO’ if the patient groups have important differences in factors that may influence the outcomes. | |  |
| 1.6 | Similar  treatment  groups | | *The only difference between groups is the treatment under investigation.*  ‘YES’ if there appears to be no important differences between treatment groups other than the treatment being studied. ‘NO’ if there appears to be an important difference between the two groups. | |  |
| 1.7 | Outcome | | *All relevant outcomes are measured in a standard, valid and reliable way.* Pre-selection condition.  ‘YES’ with use DSM validated delirium screening tools. (e.g. CAM-ICU, ICDSC) were being used in the proper cohort.  ‘NO’ DSM validated tools were used in a cohort for which it has not been validated. | |  |
| 1.8 | Drop-out | | *What percentage of the individuals or clusters recruited into each treatment arm of the study dropped out before the study was completed?*  ‘YES’ Conventionally, a 20% drop out rate is regarded as acceptable  ‘NO’ Drop-out higher than 20% | |  |
| 1.9 | Intension-to-treat | | *All the subjects are analyzed in the groups to which they were randomly allocated, referred to as intention to treat (ITT) analysis.*  ‘YES’ if ITT is mentioned in the text. ‘modified’ ITT is acceptable if an explanation is provided. ‘NO’ if ITT is not mentioned in the text. ‘Not applicable’ if all participants are accounted for and none are lost to follow-up | |  |
| 1.10 | | Comparability  of sites | | *Where the study is carried out at more than one site, results are comparable for all sites.*  ‘YES’ if there is no marked difference in the site data reported or if here is no difference in the centers that can be determined ‘NO’ if there is one or more sites that have markedly worse or better data than the others. Or if the sites have different characteristics such as community treatment against hospital in-patient treatment. ‘Can’t say’ if no site specific data is given. ‘Not applicable’ if there is only one site. | |
| Adapted from: Scottish Intercollegiate Guidelines Network. Checklist 2 – Controlled Trials. Version 2.0. Edinburgh 2012. http://www.sign.ac.uk/methodology/checklists.html | | | | |  |

| **ADDITIONAL FILE 1B**: Simplified SIGN methodology checklist of COHORT studies | | | | |
| --- | --- | --- | --- | --- |
|  | Type of bias | Applicability and consideration for our review | Final Checklist | |
| 1.1 |  | The study addresses an appropriate and clearly focused question: *Pre-selection process* | |  |
| 1.2 | Selection | *The groups studied are selected from source populations that are comparable in all aspects other than the factor under investigation.*  “YES” if random / consecutive selection of patients (with in- and exclusion criteria). | | X |
| 1.3 | Selection | *The study indicates how many of the people asked to take part in each of the groups being studied:* (Mostly) not applicable | |  |
| 1.4 | Performance | The likelihood that some subjects might have the outcome at the time of enrolment is assessed and taken into account in the analysis?  “YES” if delirium was (indirectly) assessed at time of enrolment. | | X |
| 1.5 | Attrition | *What percentage of individuals or clusters recruited into each arm of the study dropped out before the study was completed?*  Not applicable in most cohort studies due to relatively short follow-up. | |  |
| 1.6 | Attrition | *Comparison is made between full participants and those lost to follow-up, by exposure status.*  Not applicable in most cohort studies due to relatively short follow-up. | |  |
| 1.7 | Detection | The outcomes are clearly defined.  “YES” if endpoints or outcomes were clearly specified and used in analysis.  “NO” if patients have short follow-up or missing essential follow-up days. | | X |
| 1.8 | Detection | The assessment of outcome is made blind to exposure status.  “Not Applicable” when essential risk factors are possible to be blinded; e.g. age. “NO” if assessors could have been blinded for essential risk factors, but were not, e.g. cognitive / executive tests or before-after studies. | | X |
| 1.9 | Detection | *Where blinding is not possible, there is some recognition that knowledge of exposure status could have influenced the assessment of outcome.* | |  |
| 1.10 | Detection | *The measurement of exposure is reliable and if possible validated.*  *“*YES” if measurement / registration of (risk) factors was clearly defined. ”NO” if essential exposure is measured with definition or not validated*.* | | X |
| 1.11 | Detection | *Outcome assessment is validated and reliable.* Pre-selection: usage of DSM validated delirium assessment tool.  “YES” if the assessment tool was validated in the specific population; e.g. CAM-ICU instead of CAM in ventilated patients. | | X |
| 1.12 | Detection | *Exposure level or prognostic factor is assessed more than once.*  ‘Not applicable’ for most factors. “YES” if risk factors can be assessed at multiple time points, e.g. laboratory values and done. | | X |
| 1.13 /  1.14 | Statistical  Analysis | *The main potential confounders are identified and taken into account in the design and analysis. Have confidence intervals been provided?*  Pre-selection process; “YES” if essential confounders have been taken into account. “NO” if significant differences in characteristics have not been taken in to account or certain variables in the model could be consequence of delirium instead of cause. | | X |
| Adapted from: Scottish Intercollegiate Guidelines Network. Checklist 3 – Cohort studies. Version 2.0. Edinburgh 2012. http://www.sign.ac.uk/methodology/checklists.html | | | | |
