# Supplementary material for: Risk factors for delirium after on-pump cardiac surgery: a systematic review
Source: Crit Care. 2015 Sep 23;19(1):346. doi: 10.1186/s13054-015-1060-0 (PMC4579578; doi:10.1186/s13054-015-1060-0)
Supplement: Additional file 2: — Effect estimates of variables tested in multivariable analysis. (DOC 218 kb) [file 13054_2015_1060_MOESM2_ESM.doc]

**ADDITIONAL FILE 7**

Risk factors for delirium after on-pump cardiac surgery: a systematic review

A.N.C. Gosselt, MD., A.J.C. Slooter, MD., PhD., P.R.Q. Boere, MD., I.J. Zaal, MD., PhD.

| **ADDITIONAL FILE 7. Effect estimates of variables tested in multivariable analysis** | | | | | | | | | |
| --- | --- | --- | --- | --- | --- | --- | --- | --- | --- |
| **Variable** | **Study** | | **Quality** | | **OR/HR** | **(95% CI)** | **Remarks on how variable was measured** |  | |
| **Predisposing Variables** | | | | | | | | | |
| **Patient characteristics** | | | | | | | | | |
| Age | Katznelson – 2009 | | | ++ | 2.47 | (1.43-4.23) | ≥ 60 years vs <60 years | |  |
|  | Kazmierski – 2010 | | | + | 4.23 | (2.24-7.96) | ≥ 65 years vs <65 years | |  |
|  | Van der Mast – 1999 | | | + | 5.2 | (2.1-13.3) | ≥ 65 years vs <65 years | |  |
|  | Arenson - 2013 | | | - | 3.21 | (2.02-5.10) | ≥ 65 years vs <65 years | |  |
|  | Schoen – 2011 | | | ++ | 4.30 | (1.54-12.04) | ≥ 70 years vs < 70 years | |  |
|  | Santana-Santos – 2004 | | | + | 1.10 | (1.03-1.18) | Per year increase | |  |
|  | Sauer – 2014 | | | ++ | 1.08 | (1.06-1.11) | Per year increase | |  |
|  | Palmbergen – 2012 | | | - | 1.06 | (1.02-1.11) | Per year increase | |  |
|  | Taipale – 2012 | | | ++ | 1.07 | (1.02-1.12) | Per year increase | |  |
|  | Mariscalco – 2012 | | | ++ | 1.04 | (1.01-1.07) | Per year increase | |  |
|  | Roggenbach – 2014 | | | ++ | 1.16 | (1.04-1.29) | Per year increase | |  |
|  | Afonso – 2010 | | | ++ | 2.5 | (1.6-3.9) | Per 10 year increase | |  |
|  | Maldonado – 2009 | | | + | 1.3 | (1.1-1.5) | Per 10 year increase | |  |
| Opium use | Eizadi-Mood – 2014 | | | + | 0.06 | (0.037-0.13) | Preadmission frequent usage of opiods (N/Y) | |  |
| Nicotine Use | Roggenbach – 2014 | | | ++ | 32.37 | (3.9-268) | Preadmission current usage of tobacco (Y/N) | |  |
|  | Santana-Santos – 2004 | | | + | 3.33 | (1.19-9.33) | Preadmission current usage of tobacco (Y/N) | |  |
| **Chronic Pathology** | | | | | | | | | |
| Hypertension | Mariscalco – 2012 | | | ++ | 1.83 | (1.14-2.92) | Preadmission comorbidity in medical history (Y/N) | |  |
|  | Santana-Santos – 2004 | | | + | 3.40 | (1.34-8.68) | Preadmission comorbidity in medical history (Y/N) | |  |
| COPD | Mariscalco – 2012 | | | ++ | 1.99 | (1.12-3.54) | Preadmission comorbidity in medical history (Y/N) | |  |
| Diabetes Mellitus | Smulter – 2013 | | | + | 3.24 | (1.13-9.32) | Preadmission comorbidity in medical history (Y/N) | |  |
| History of CVA | Mariscalco – 2012 | | | ++ | 2.14 | (1.13-4.06) | Preadmission comorbidity in medical history (Y/N) | |  |
|  | Sauer – 2014 | | | ++ | 1.90 | (1.05-3.43) | Preadmission comorbidity in medical history (Y/N) | |  |
| Atherosclerosis | Rudolph – 2005 | | | + | 2.7 | (1.1-6.8) | RR per 1 point increase in atherosclerosis score. | |  |
| Periferal vascular disease | Taipale – 2012 | | | ++ | 4.52 | (1.31-15.59) | Preadmission comorbidity in medical history (Y/N) | |  |
| Hyperlipidaemie | Eizadi-Mood – 2014 | | | + | 0.34 | (0.19-0.64) | Preadmission comorbidity in medical history (N/Y) | |  |
| Atrial fibrillation | Kazmierski – 2010 | | | + | 3.67 | (1.40-9.60) | Preadmission comorbidity in medical history (Y/N) | |  |
| Neurological / psychiatric | Schoen – 2011 | | | ++ | 6.22 | (2.02-19.16) | Preadmission comorbidity in medical history (Y/N) | |  |
| Risk Score | Hakim – 2012 | | | ++ | 2.62 | (1.51-4.53) | Per 1 point increase Rudolph Risk Score. | |  |
|  | Palmbergen – 2012 | | | - | 1.41 | (1.17-1.71) | Not validated Risk Score | |  |
| Depression | Katznelson – 2009 | | | ++ | 3.06 | (1.36-6.90) | Preadmission comorbidity in medical history / referral letter (Y/N) | |  |
|  | Kazmierski – 2013 ^f^ | | | ++ | 15.8 | (1.66-150.5) | Definition according Mini International Neuropsychiatric Interview | |  |
|  | Kazmierski – 2014 (A) ^f^ | | | ++ | 10.9 | (1.68-70.67) | Definition according Mini International Neuropsychiatric Interview | |  |
| ^¥^ not described whether measured before delirium onset.  ^a^ not significant but provided multivariable point estimate ≤ 0.5 or ≥ 1.5, ^b^ not specified determination of TIA/stroke, ^c^ not significant but tested in randomised controlled trial, ^d^ Significant reduction in delirium duration, ^e^ point estimates of multivariable analysis. ^f^ = same study cohort, counted as 1. ^g^ = CI as mentioned in article seems not correct.  CI = Confidence Interval, COPD = Chronic Obstructive Pulmonary Disease, CVA = Cerebrovascular Accident, FFP = Fresh frozen plasma, HR= Hazard Ratio, RBC = Red Blood cell Concentrate, RIFLE = Risk, Injury, Failure, Loss of function, and End-stage renal disease, OR= Odds Ratio | | | | | | | | | |
| **ADDITIONAL FILE 7. *(continued)* Effect estimates of variables tested in multivariable analysis** | | | | | | | | | |
| **Variable** | **Study** | | **Quality** | | **OR/HR** | **(95% CI)** | **Remarks on how variable was measured** |  | |
| **Predisposing Variables** | | | | | | | | | |
| **Chronic Pathology** | | | | | | | | | |
|  | Kazmierski – 2010 | | | + | 4.69 | (1.84-11.93) | Definition according Mini International Neuropsychiatric Interview | |  |
|  | Tully – 2010 | | | + | 3.86 | (1.42-10.52) | Definition according Mini International Neuropsychiatric Interview / DSM | |  |
|  | Van der Mast – 1999 | | | + | 2.0^a^ | (0.9-4.2) | General Health Questionnaire score >7 vs ≤7 | |  |
| Anxiety disorder | Tully – 2010 | | | + | 1.71^a^ | (0.47-6.27) | Definition according Mini International Neuropsychiatric Interview / DSM | |  |
| Panic disorder | Tully – 2010 | | | + | 1.73^a^ | (0.43-6.88) | Definition according Mini International Neuropsychiatric Interview / DSM | |  |
| Cognitive dysfunction | Kazmierski – 2013 ^f^ | | | ++ | 1.02 | (1.01-1.03) | Trial Making Test part B score, per point | |  |
|  | Kazmierski – 2014 (A) ^f^ | | | + | 6.33 | (31.9-20.8)^g^ | Mild Cognitive Impairment preadmission according to MoCA screening (Y/N) | |  |
|  | Kazmierski – 2014 (A) ^f^ | | | + | 22.96 | (2.14-246.7) | Dementia preadmission comorbidity according to MoCA screening (Y/N) | |  |
|  | Kazmierski – 2010 | | | + | 6.14 | (3.31-11.39) | Mini mental state examination <25 vs ≥25 | |  |
|  | Veliz-Reissmuller – 2007 | | | + | 11.3 | (2.7-47.7) | Mini mental state examination ≤28 vs ≥29 | |  |
|  |  |  |  |  | 3.37 | (1.00-11.5) | Subjective memory complaints in previous 6 months vs no complaints | |  |
|  | Rudolph – 2006 | | | + | 2.77 | (1.12-6.87) | RR group ≥0.5 SD below composite executive functioning vs ≤ 0.5 above mean | |  |
|  | Rudolph – 2006 | | | + | 0.49^a^ | (0.19-1.25) | RR per 0.5 SD below ≥0.5 SD below mean composite memory functioning vs ≤ 0.5 | |  |
|  | Schoen – 2011 | | | ++ | 2.23 | (0.76-6.52) | Mini mental state examination 24-27 vs >27 | |  |
|  | | | |  | 6.50 | (1.75-24.13) | Mini mental state examination ≤23 vs >27 | |  |
| Frailty | Jung – 2014 | | | ++ | 5.05 | (1.58-16.1) | Modified Fried Criteria ≥3 (out of 7) versus <3 | |  |
|  |  | | | ++ | 8.26 | (2.23-30.6) | Short Physical Performance Battery score 4-6 | |  |
|  |  | | | ++ | 3.72 | (1.39-9.92) | Frailty Index Score ≥0.3 | |  |
| Type D personality | Tully – 2010 |  | | + | 2.85^a^ | (0.97-8.38) | According to DS14 (Y/N) | |  |
| Preoperative pain score | Smulter – 2013 | | | + | 1.20 | (1.01-1.44) | Per point increase op preoperative NRS-pain scale | |  |
| **Preoperative diagnostics** | | | | | | | | | |
| Leucocytes | Eizadi-Mood – 2014 | | | + | 0.62 | (0.44-0.87) | Per 10^3^/mL increase | |  |
| Cortisol | Kazmierski 2013 | | | ++ | 1.005 | (1.001-1.009) | Per 1 nmol/L serum cortisol increase | |  |
| Urea | Eizadi-Mood – 2014 | | | + | 1.22 | (1.02-1.46) | Per mg/dL increase | |  |
|  | Santana-Santos – 2004 | | | + | 1.03 | (1.00-1.05) | 1 mg/dl serum urea increase | |  |
| Creatinine | Katznelson – 2009 | | | ++ | 2.96 | (1.9-4.63) | Preoperative creatinine >150mM | |  |
| Anemia | Kazmierski – 2010 | | | + | 4.77 | (1.35-16.82) | Preoperative anemia vs no anemia (not further defined) | |  |
|  | Tully – 2010 | | | + | 1.69 | (1.24-2.29) | Preoperative hemoglobin (not further defined) | |  |
| Albumin | Van der Mast – 1999 | | | + | 3.5 | (1.6-7.6) | ≤40g/L vs >40g/L | |  |
| Triglycerides | Eizadi-Mood – 2014 | | | + | 1.009 | 1.003-1.014) | Per ? increase | |  |
| LVEF% | Mariscalco – 2012 | | | ++ | 1.55 | (0.96-2.4) | Left Ventricular Ejection Fraction 30-50% vs >50% | |  |
|  |  | | |  | 2.74 | (1.39-5.28) | Left Ventricular Ejection Fraction <30% vs >50% | |  |
| Oxygen saturation | Smulter – 2013 | | | + | 0.82 | (0.67-1.00) | Per percent increase in peripheral oxygen saturation | |  |
|  | Schoen – 2011 | | | ++ | 3.27 | (1.14-9.37) | Regional cerebral oxygen saturation ≤59.5% vs >59.5% | |  |
| ^¥^ not described whether measured before delirium onset.  ^a^ not significant but provided multivariable point estimate ≤ 0.5 or ≥ 1.5, ^b^ not specified determination of TIA/stroke, ^c^ not significant but tested in randomised controlled trial, ^d^ Significant reduction in delirium duration, ^e^ point estimates of multivariable analysis. ^f^ = same study cohort, counted as 1.  CI = Confidence Interval, COPD = Chronic Obstructive Pulmonary Disease, CVA = Cerebrovascular Accident, FFP = Fresh frozen plasma, HR= Hazard Ratio, RBC = Red Blood cell Concentrate, RIFLE = Risk, Injury, Failure, Loss of function, and End-stage renal disease, OR= Odds Ratio | | | | | | | | | |
| **ADDITIONAL FILE 7. *(continued)* Effect estimates of variables tested in multivariable analysis** | | | | | | | | | |
| **Variable** | **Study** | | **Quality** | | **OR/HR** | **(95% CI)** | **Remarks on how variable was measured** |  | |
| **Predisposing Variables** | | | | | | | | | |
| **Preoperative diagnostics** | | | | | | | | | |
| Apnea-hypopnea Index | Roggenbach – 2014 | | | ++ | 1.05 | (1.01-1.10) | Per 1 point increase on the preoperative apnea-hypopnea index | |  |
| Cardiothoracic Index | Santana-Santos – 2004 | | | + | 2.93 | (1.33-6.47) | >50% vs <50% on X-thorax | |  |
| Ratio Phe:oLNAA | Mast – 1999 | | | + | 2.5^a^ | (0.9-6.5) | Phenylalanine to ((iso-)leucine, valine, tyrosine, tryptophan) ≥13.35 | |  |
| **Preoperative Medication** | | | | | | | | | |
| Statin | Katznelson – 2009 | | | ++ | 0.54 | (0.35-0.84) | Statin vs no statin usage | |  |
|  | Mariscalco – 2012 | | | ++ | 1.52^a^ | (0.97-2.37) | Statin vs no statin usage | |  |
| Nifedipine | Van der Mast – 1999 | | | + | 2.4 | (1.0-5.8) | Preoperative use of nifedipine (Y/N) | |  |
| Benzodiazepine | Arenson – 2013 | | | - | 2.11 | (1.09-4.07) | Pre-admission to the hostpital use of benzodiazepine (Y/N) | |  |
| Psychotrophic | Tully – 2010 | | | + | 6.17 | (1.27-30.12) | Psychotrophic or anti-cholinergic drugs (Y/N) | |  |
| ^¥^ not described whether measured before delirium onset.  ^a^ not significant but provided multivariable point estimate ≤ 0.5 or ≥ 1.5, ^b^ not specified determination of TIA/stroke, ^c^ not significant but tested in randomised controlled trial, ^d^ Significant reduction in delirium duration, ^e^ point estimates of multivariable analysis. ^f^ = same study cohort, counted as 1.  CI = Confidence Interval, COPD = Chronic Obstructive Pulmonary Disease, CVA = Cerebrovascular Accident, FFP = Fresh frozen plasma, HR= Hazard Ratio, RBC = Red Blood cell Concentrate, RIFLE = Risk, Injury, Failure, Loss of function, and End-stage renal disease, OR= Odds Ratio | | | | | | | | | |

| **ADDITIONAL FILE 7. (*Continued*). Effect estimates of variables tested in multivariable analysis** | | | | | | | | | |
| --- | --- | --- | --- | --- | --- | --- | --- | --- | --- |
| **Variable** | **Study** | **Quality** | | **OR/HR** | | **(95% CI)** |  |  | |
| **Precipitating Variables** | | | | | | | | | |
| **Type of surgery** | | | | | | | | | |
| CABG and valve | Arenson – 2013 | | - | 2.3 | | (1.36-3.88) | Coronary artery bypass graft+valve vs rest | |  |
|  | Katznelson – 2009 | | ++ | 1.86 | | (1.16-2.98) | Coronary artery bypass graft +valve vs rest | |  |
|  | Mariscalco – 2012 | | ++ | 3.42 | | (2.17-5.37) | Coronary artery bypass graft +valve vs Coronary artery bypass graft | |  |
|  | Veliz-Reismuller – 2007 | | + | 3.25^a^ | | (0.8-13.0) | Coronary artery bypass graft +valve vs Coronary artery bypass graft | |  |
|  |  |  |  | 3.90 | | (1.0-15.8) | Valve vs Coronary artery bypass graft | |  |
|  | Palmbergen – 2012 | | - | 3.68 | | (1.77-7.66) | Coronary artery bypass graft+ vs Coronary artery bypass graft | |  |
|  | Smulter – 2013 | | + | 2.98 | | (1.15-7.70) | Combined vs single procedure | |  |
| **Perioperative** | | | | | | | | | |
| IABP support | Katznelson – 2009 | | ++ | 3.84 | | (1.72-8.56) | Support of Intra-aortic balloon (Y/N) | |  |
| Blood transfusion | Santana-Santos – 2004 | | + | 1.00 | (1.000-1.002) | | Per ml blood replacement intra-operatively | |  |
|  | Katznelson – 2009 | | ++ | 3.3 | | (2.09-5.19) | Perioperative transfusion with > 5 units RBC vs ≤ 5units | |  |
|  | Arenson – 2013 | | - | 2.02 | | (1.30-3.15) | Postoperative transfusion of any blood product (RBC/FFP/platelets) vs none | |  |
| **Intraoperative** | | | | | | | | | |
| Hemoglobin | Kazmierski – 2014 (A) | | + | 0.72 | | (0.54-0.98) | Per 1mg/dL increase. | |  |
| Length of surgery | Afonso – 2010 | | ++ | 1.3 | | (1.1-1.5) | Per 30minutes increase | |  |
| ACC-time | Tully – 2010 | | + | 1.02 | | (1.0-1.04) | Aortic Cross Clamp time per minute increase | |  |
| Reperfusion time | Kazmierski – 2014 (B) | | ++ | 1.10 | | (1.02-1.19) | Not described | |  |
| Lowest temperature | Detroyer – 2008 | | ++ | 0.86 | | (0.74-0.99) | Not described | |  |
| Microembolic load | Rudolph – 2009 | | + | 0.5^a^ | | (0.2-1.4) | Per tertile increase in amount of micro embolism. | |  |
| Midazolam dose | Kazmierski – 2014 (B) ^f^ | | ++ | 1.1 | | (1.03-1.17) | Per mg increase | |  |
|  | Kazmierski – 2013 ^f^ | | ++ | 1.08 | | (1.03-1.15) | Per mg increase | |  |
| Blood transfusion | Roggenbach – 2014 | | ++ | 1.55 | | (1.03-2.32) | Per unit blood transfusion intraoperative. | |  |
| Fentanyl | Burkhart – 2010 | | ++ | 4.9 | | (1.72-13.8) | Per 10 µg/kg increase | |  |
| **Postoperative Variables** | | | | | | | | | |
| Stroke or TIA^b / ¥^ | Arenson – 2013 | | - | 6.03 | | (1.69-21.53) | Postoperative stroke or Transien Ischemic Accident (Y/N) | |  |
| Cardiac arrhythmia ^¥^ | Kazmierski – 2014 (B) ^f^ | | ++ | 8.77 | | (1.04-73.88) | Postoperative Atrial fibrillation according to 24-hour ElectroCardiogram (Y/N) | |  |
|  | Kazmierski – 2014 (A)^f^ | | + | 7.03 | | (1.5-32.2) | Postoperative Atrial fibrillation according to 24-hour ElectroCardiogram (Y/N) | |  |
|  | Mariscalco – 2012 | | ++ | 1.88 | | (1.19-2.95) | Postoperative Atrial fibrillation (Y/N) | |  |
| Renal insufficiency ^¥^ | Prakanrattana – 2007 | | + | 13.89^a^ | | (0.99-197.3) | Elevation of creatinine level necessitating renal replacement therapy (Y/N) | |  |
|  | Arenson – 2013 | | - | 1.91 | | (1.08-3.37) | >50% increase from baseline creatinine level | |  |
|  | Mariscalco –2012 | | + | 3.07 | | (1.87-5.05) | Acute kidney injury according to RIFLE criteria. (Y/N) | |  |
| Acute infection ^¥^ | Chang – 2008 | | - | 6.9 | | ? | No definition given | |  |
| Cardiogenic shock ^¥^ | Chang – 2008 | | - | 2.75 | | ? | No definition given | |  |
| ^¥^ not described whether measured before delirium onset.  ^a^ not significant but provided multivariable point estimate ≤ 0.5 or ≥ 1.5, ^b^ not specified determination of TIA/stroke, ^c^ not significant but tested in randomised controlled trial, ^d^ Significant reduction in delirium duration, ^e^ point estimates of multivariable analysis. ^f^ = same study cohort, counted as 1.  CI = Confidence Interval, COPD = Chronic Obstructive Pulmonary Disease, CVA = Cerebrovascular Accident, FFP = Fresh frozen plasma, HR= Hazard Ratio, RBC = Red Blood cell Concentrate, RIFLE = Risk, Injury, Failure, Loss of function, and End-stage renal disease, OR= Odds Ratio | | | | | | | | | |
| **ADDITIONAL FILE 7. (*Continued*). Effect estimates of variables tested in multivariable analysis** | | | | | | | | | |
| **Variable** | **Study** | **Quality** | | **OR/HR** | | **(95% CI)** |  |  | |
| **Precipitating Variables** | | | | | | | | | |
| **Postoperative Variables** | | | | | | | | | |
| IL-2 ^¥^ | Kazmierski – 2013 ^f^ | | ++ | 1.002 | | (1.000-1.004) | Interleukin-2 on first postoperative day per U/ml increase | |  |
|  | Kazmierski – 2014 (B) ^f^ | | ++ | 1.002 | | (1.000-1.004) | Interleukin-2 on first postoperative day per U/ml increase | |  |
| Highest CRP^¥^ | Burkhart – 2010 | | ++ | 1.10 | | (1.01-1.16) | C-reactive protein per 10-mg/L increase | |  |
| Albumin ^¥^ | Chang – 2008 | | - | 2.4 | | ? | Postoperative <3.0g/dL vs ≥3.0g/dL | |  |
| Urea^¥^ | Eizadi-Mood – 2014 | | + | 1.15 | | (1.017-1.31) | Per mg/dL increase first day postoperative | |  |
| Creatinine^¥^ | Eizadi-Mood – 2014 | | + | 0.08 | | (0.006-0.96) | Per mg/dL increase first day postoperative | |  |
| Haematocrit^¥^ | Chang – 2008 | | - | 2.16 | | (1.16-4.03) | Postoperative <30% vs >30%. | |  |
| Fasting blood glucose^¥^ | Eizadi-Mood – 2014 | | + | 1.02 | | (1.008-1.03) | Per ? increase on the first day postoperative | |  |
| Hypoxia^¥^ | Kazmierski – 2010 | | + | 3.24 | | (1.77-5.94) | pO2 <60 mmHg (duration not described) | |  |
|  | Prakanrattana – 2007 | | + | 13.78 | | (1.2-165.2) | Respiratory failure (hypoxia/hypercarbia) vs no respiratory failure (not further defined) | | |
|  | Kazmierski – 2014 (A) | | + | 5.65 | | (1.88-16.97) | pO2 <60 mmHg (duration not described) | |  |
| Prolonged MV^¥^ | Norkiene – 2013 | | - | 1.15 | | (1.02-1.28) | Per hour increase in mechanical ventilation | |  |
|  | Burkhart – 2010 | | ++ | 1.1 | | (1.04-1.21) | Per hour increase | |  |
|  | Detroyer – 2008 | | ++ | 1.1 | | (1.05-1.15) | Not described | |  |
|  | Arenson – 2013 | | - | 4.34 | | (2.52-7.45) | >24hours vs ≤ 24hours | |  |
|  | Kazmierski – 2010 | | + | 5.29 | | (2.14-13.06) | >24hours vs ≤ 24hours | |  |
| Time from opening eyes to following commands | Prakanrattana – 2007 | | + | 4.57 | | (1.66-12.59) | >70min vs. ≤70min | |  |
| ^¥^ not described whether measured before delirium onset.  ^a^ not significant but provided multivariable point estimate ≤ 0.5 or ≥ 1.5, ^b^ not specified determination of TIA/stroke, ^c^ not significant but tested in randomised controlled trial, ^d^ Significant reduction in delirium duration, ^e^ point estimates of multivariable analysis. ^f^ = same study cohort, counted as 1. CI = Confidence Interval, COPD = Chronic Obstructive Pulmonary Disease, CVA = Cerebrovascular Accident, FFP = Fresh frozen plasma, HR= Hazard Ratio, RBC = Red Blood cell Concentrate, RIFLE = Risk, Injury, Failure, Loss of function, and End-stage renal disease, OR= Odds Ratio | | | | | | | | | |

| **ADDITIONAL FILE 7. (*Continued*). Effect estimates of variables tested in multivariable analysis** | | | | | | | |
| --- | --- | --- | --- | --- | --- | --- | --- |
| **Variable** | **Study** | **Quality** | | **OR/HR** | **(95% CI)** |  | |
| Protecting variables | | | | | | | |
| Additional Screening | Palmbergen – 2012 | | - | 0.37 | (0.16-0.86) | Haga Brain Care Strategy vs. no additional screening + consequential intervention |  |
| **Perioperative medication** | | | | | | | |
| Ketamin | Hudetz – 2009 | | ++ | 12.6 | (1.5-107.5) | Placebo vs Ketamine (5mg/kg) bolus with anesthetic induction |  |
| Dexamethasone | Sauer – 2014 | | ++ | 0.95^c^ | (0.63-1.43) | Dexamethason 1mg/kg at anesthetic induction vs placebo |  |
| Risperidone | Prakanrattana – 2007 | | ++ | 0.35 | (0.16-0.77) | RR for treatment with Risperidone (1mg) vs placebo at regaining consciousness | |
|  | Hakim – 2012 | | ++ | 3.83 | (1.63-8.98) | Subhazard ratio for placebo vs Risperidone (0.5mg 2d) for treatment of SSD. |  |
| Dexmedetomidine | Shehabi – 2009 | | +^c/d^ | 0.57 | (0.26-1.10) | Incidence delirium 8.6% Dexmedetomidine vs 15% in morphine-group. |  |
|  | Park – 2014 | | + | 0.34 | (0.12-0.91) | Incidence delirium 9.0% Dexmedetomidine vs 22.7% in Remifentanil-group. |  |
|  | Maldonado – 2009 | | - | 28.6^e^ | (4.7-262.5) | Midazolam vs. Dexmedetomidine at Intensive Care Unit arrival |  |
|  |  |  | - | 29.6^e^ | (4.8-280.6) | Propofol vs. Dexmedetomidine at Intensive Care Unit arrival |  |
| ^¥^ not described whether measured before delirium onset.  ^a^ not significant but provided multivariable point estimate ≤ 0.5 or ≥ 1.5, ^b^ not specified determination of TIA/stroke, ^c^ not significant but tested in randomised controlled trial, ^d^ Significant reduction in delirium duration, ^e^ point estimates of multivariable analysis. ^f^ = same study cohort, counted as 1.  CI = Confidence Interval, COPD = Chronic Obstructive Pulmonary Disease, CVA = Cerebrovascular Accident, FFP = Fresh frozen plasma, HR= Hazard Ratio, RBC = Red Blood cell Concentrate, RIFLE = Risk, Injury, Failure, Loss of function, and End-stage renal disease, OR= Odds Ratio | | | | | | | |
