# Supplementary material for: Risk factors for delirium after on-pump cardiac surgery: a systematic review
Source: Crit Care. 2015 Sep 23;19(1):346. doi: 10.1186/s13054-015-1060-0 (PMC4579578; doi:10.1186/s13054-015-1060-0)
Supplement: Additional file 3: — List of articles screened in full text review and the rationale for inclusion or exclusion. (DOC 216 kb) [file 13054_2015_1060_MOESM3_ESM.doc]

**ADDITIONAL FILE 2**

Risk factors for delirium after on-pump cardiac surgery: a systematic review

A.N.C. Gosselt, MD., A.J.C. Slooter, MD., PhD., P.R.Q. Boere, MD., I.J. Zaal, MD., PhD.

| **ADDITIONAL FILE 2: List of articles screened in full-text review and the rationale for inclusion or exclusion** | | | | | | | | | | | | | | | | | | | | |
| --- | --- | --- | --- | --- | --- | --- | --- | --- | --- | --- | --- | --- | --- | --- | --- | --- | --- | --- | --- | --- |
| **Author** | **Reference** | **Exclusion reason(s)** | | | | | | | | | | | | | | |  | | |  |
|  |  | **Study population** | | **Delirium (Assessment)** | | | | | **Outcome**  **(incl. analysis)** | | | | | | | **Other** | **Remarks** | | | **Inclusion** |
| Afonso,A. | Semin.Cardiothorac.Vasc.Anesth. 2010; 14(3):212-217 |  | | |  | | | | |  | | |  | | | |  | | | V |
| Ahigren,E. | J.Cardiothorac.Vasc.Anesth. 1998; 12(3):270-273 | X | | | X | | | | | X | | |  | | | |  | | |  |
| Albacker,T. B. | Interact.Cardiovasc.Thorac.Surg. 2009; 9(1):56-60 | X | | | X | | | | | X | | |  | | | |  | | |  |
| Andrejaitiene,J. | Perfusion 2012; 27 (2):105-112 | X | | |  | | | | | X | | |  | | | | Mild vs severe delirium | | |  |
| Arenson,B. G. | J.Thorac.Cardiovasc.Surg. 2013; 146(1):172-178 |  | | |  | | | | |  | | |  | | | |  | | | V |
| Astaneh,A. N. | Pak.J.Med.Sci. 2007; 23(2):188-192 | X | | | X | | | | | X | | |  | | | |  | | |  |
| Bakker,R. C. | Eur.J.Cardiothorac.Surg. 2012; 41(3):544-549 | X | | |  | | | | |  | | |  | | | |  | | |  |
| Banach,M. | Med.Sci.Monit. 2008; 14(5):286-291 |  | | |  | | | | |  | | | X | | | | Partly cohort Kazmierski 2010 | | |  |
| Baranyi,A. | Psychosomatics 2014 |  | | |  | | | | | X | | |  | | | |  | | |  |
| Baranyi,A. | Brain Inj. 2013; 27(4):417-424 |  | | |  | | | | | X | | |  | | | |  | | |  |
| Baranyi,A. | Psychiatry Res. 2012; 200(2):957-963 |  | | |  | | | | | X | | |  | | | |  | | |  |
| Boef,A. G. C. | Epidemiology 2014;25: 923–927 | X | | | X | | | | |  | | |  | | | |  | | |  |
| Brown,C. H. | Anesth. Analg. 2014;119(5):1011-1013 |  | | |  | | | | |  | | | X | | | | Editorial | | |  |
| Brown,C. H. | Anesth. Analg. 2014;119(2):242-250 |  | | |  | | | | |  | | | X | | | | Case-control study | | |  |
| Bucerius,J. | J.Thorac.Cardiovasc.Surg. 2004; 127(1):57-64 | X | | | X | | | | |  | | |  | | | |  | | |  |
| Bucerius,J. | Z.Kardiol. 2005; 94(9):575-582 | X | | | X | | | | |  | | |  | | | |  | | |  |
| Burkhart,C. S. | J.Cardiothorac.Vasc.Anesth. 2010; 24(4):555-559 |  | | |  | | | | |  | | |  | | | |  | | | V |
| Burns, K.D. | BCMJ 2009; 51(5):206-210 | X | | | X | | | | |  | | |  | | | |  | | |  |
| Chang,Y. L. | Am.J.Crit.Care 2008; 17(6):567-575 |  | | |  | | | | |  | | |  | | | |  | | | V |
| Christenson,J. T. | Coron.Artery Dis. 1994; 5(2):169-174 |  | | | X | | | | | X | | |  | | | |  | | |  |
| Clark,R. E. | J.Thorac.Cardiovasc.Surg. 1995; 109(2):249-57 |  | | | X | | | | | X | | |  | | | |  | | |  |
| Cruz,J. N. | Rev.Bras.Ter.Intensiva 2012; 24(1):52-57 | X | | |  | | | | | X | | |  | | | |  | | |  |
| Detroyer,E. | J.Am.Geriatr.Soc. 2008; 56(12):2278-2284 |  | | |  | | | | |  | | |  | | | |  | | | V |
| Dong,S. | Peptides 59 (2014) 70–74 | X | | |  | | | | |  | | |  | | | |  | | |  |
| Eizadi-Mood,N. | Int J Prev Med. 2014 Jul; 5(7): 900–906. |  | | |  | | | | |  | | |  | | | |  | | | V |
| Eriksson,M. | Scand.Cardiovasc.J. 2002; 36(4):250-255 |  | | |  | | | | | X | | |  | | | |  | | |  |
| Ebert,A.D. | J.Cardiothorac.Vasc.Anesth. 2001; 15(1):15-19 |  | | | X | | | | | X | | |  | | | |  | | |  |
| Finkelmeier,B. A. | J.Cardiovasc.Nurs. 1993; 7(4):38-46 |  | | | X | | | | | X | | |  | | | |  | | |  |
| Gamberini,M. | Crit.Care Med. 2009; 37(5):1762-1768 |  | | |  | | | | |  | | |  | | | |  | | | V |
| Gandhi,G. Y. | Mayo Clin.Proc. 2005; 80(7):862-866 | X | | | X | | | | | X | | |  | | | |  | | |  |
| SUBTOTAL |  | 12 | | | 12 | | | | | 14 | | | 3 | | | |  | | | 7 |
| **ADDITIONAL FILE 2: *(Continued)* List of articles screened in full-text review and the rationale for inclusion or exclusion** | | | | | | | | | | | | | | | | | | | | |
|  |  | **Exclusion reason(s)** | | | | | | | | | | | | | | | |  | |  |
| **Author** | **Reference** | **Study population** | | | | **Delirium (Assessment)** | | | | | **Outcome**  **(incl. analysis)** | | | **Other** | | | | **Remarks** | | **Inclusion** |
| Gasparovic,H. | Vasc.Med. 2013; 18(5):275-281 |  | | | | X | | | | | X | | |  | | | |  | |  |
| Giltay,E. J. | Eur.J.Cardiothorac.Surg. 2006; 30(1):140-147 | X | | | | X | | | | |  | | |  | | | |  | |  |
| Gokgoz,L. | Scand.Cardiovasc.J. 1997; 31(4):217-222 |  | | | | X | | | | | X | | |  | | | |  | |  |
| Gottesman,R. F. | Ann.Neurol. 2010; 67(3):338-344 | X | | | | X | | | | |  | | |  | | | |  | |  |
| Grega,M. A. | Ann.Thorac.Surg. 2003; 75(5v:1387-1391 |  | | | | X | | | | |  | | |  | | | |  | |  |
| Guenther,U. | Ann.Surg. 2013; 257(6):1160-1167 | X | | | |  | | | | |  | | |  | | | |  | |  |
| Hakim,S.M. | Anaesthesia 2012; 116(5) 987-997 |  | | | |  | | | | |  | | |  | | | |  | | V |
| Hansdottir,V. | Anesthesiology 2006; 104(1):142-151 |  | | | | X | | | | | X | | |  | | | |  | |  |
| Hatano,Y. | Am.J.Geriatr.Psychiatry 2013; 21(10):938-945 | X | | | | X | | | | |  | | |  | | | |  | |  |
| Heijmeriks,J. A. | Clin.Cardiol. 2000; 23(7):540-546 |  | | | | X | | | | | X | | |  | | | |  | |  |
| Herrmann,M. | Eur.J.Cardiothorac.Surg. 1999; 16(5):513-518 |  | | | | X | | | | | X | | |  | | | |  | |  |
| Hori.D. | Brit.Jour.Anaesth. 2014;113 (6): 1009–17 |  | | | | X | | | | |  | | |  | | | |  | |  |
| Hudetz,J. A. | J.Cardiothorac.Vasc.Anesth. 2010; 24(4):560-567 |  | | | |  | | | | | X | | |  | | | |  | |  |
| Hudetz,J. A. | J.Cardiothorac.Vasc.Anesth. 2011; 25(5):811-816 |  | | | |  | | | | | X | | |  | | | |  | |  |
| Hudetz,J. A. | J.Cardiothorac.Vasc.Anesth. 2009; 23(5):651-657 |  | | | |  | | | | |  | | |  | | | |  | | V |
| Jannati,Y. | Oman Medical Journal (2014) Vol. 29, No. 5:340-345 |  | | | |  | | | | | X | | |  | | | |  | |  |
| Ji,F. | Circulation 2013; 127(15):1576-1584 |  | | | | X | | | | |  | | |  | | | |  | |  |
| Ji,F. | J.Cardiothorac.Vasc.Anesth. 2014; 28(2):267–273 |  | | | | X | | | | |  | | |  | | | |  | |  |
| Jodati,A. | J.Cardiovasc.Thorac.Res.2013; 5(3):97-99 | X | | | | X | | | | | X | | |  | | | |  | |  |
| Jung,P. | J.Thorac.Cardiovasc Surg 2014;-:1-7 |  | | | |  | | | | |  | | |  | | | |  | | V |
| Karlidag,R. | Gen.Hosp.Psychiatry 2006; 28(5):418-423 | X | | | |  | | | | | X | | |  | | | |  | |  |
| Katznelson,R. | Anesthesiology 2009; 110(1):67-73 |  | |  | | | | |  | | | | | |  | | |  | | V |
| Kazmierski,J. (A) | Dement Geriatr Cogn Disord 2014;38:65–78 |  | |  | | | | |  | | | | | |  | | | } | Same cohort | V |
| Kazmierski,J. (B) | Internat. Psychogeriatrics 2014, 26:5, 845–855 |  |  | | | | |  | | | | | | |  |  |  |  |  | V |
| Kazmierski,J. | Crit.Care 2013; 17(2):38 |  |  | | | | |  | | | | | | |  |  |  |  |  | V |
| Kazmierski,J. | J.Psychosom.Res. 2010; 68(6):636- |  | |  | | | | |  | | | | | | |  | |  | | V |
| Kazmierski,J. | Gen.Hosp.Psychiatry 2006; 28(6):536-538 |  | | | |  | | | | |  | | | X | | | | Partly cohort Kazmierski 2010 | |  |
| Klugkist,M. | Anaesthesist 2008; 57(5):464-474 |  | | | |  | | | | | X | | |  | | | |  | |  |
| Kobayashi,T. | Jpn.J.Thorac.Cardiovasc.Surg. 2002; 50(4):152-157 |  | | | | X | | | | | X | | |  | | | |  | |  |
| SUBTOTAL |  | 6 | | 14 | | | | | 13 | | | | | | | 1 | |  | | 8 |
| **ADDITIONAL FILE 2: *(Continued)* List of articles screened in full-text review and the rationale for inclusion or exclusion** | | | | | | | | | | | | | | | | | | | | |
|  |  | **Exclusion reason(s)** | | | | | | | | | | | | | | | |  | |  |
| **Author** | **Reference** | **Study population** | | | | **Delirium (Assessment)** | | | | | **Outcome**  **(incl. analysis)** | | | **Other** | | | | **Remarks** | | **Inclusion** |
| Koster,S. | Ann.Thorac.Surg. 2008;86:1883-1887 | X | | | |  | | | | |  | | |  | | | |  | |  |
| Koster,S. | Eur.J.Cardiovasc.Nurs. 2013; 12(3):284-292 | X | | | |  | | | | |  | | |  | | | |  | |  |
| Kowalczuk,A. | Postepy Kardiol.Interwencyjnej 2012; 8(1):14-17 |  | | | | X | | | | | X | | |  | | | |  | |  |
| Krzych,L.J. | Canad.J.Card. 2014; 30: 932-939 | X | | | | X | | | | |  | | |  | | | |  | |  |
| Krzych,L.J. | Biomed.Res.Int. 2013; ID# 835850 | X | | | |  | | | | |  | | |  | | | |  | |  |
| Li,H-C. | Jour.CardioVasc.Nurs. (epub ahead of date) | X | | | |  | | | | | X | | |  | | | |  | |  |
| Loponen,P. | Scand.Cardiovasc.J. 2008; 42(5):337-344 | X | | | | X | | | | |  | | |  | | | |  | |  |
| Maldonado,J. R. | Psychosomatics 2009; 50(3):206-217 |  | | | |  | | | | |  | | |  | | | |  | | V |
| Mardani,D. | J.Res.Med.Sci. 2013; 18(2):137-143 |  | | | | X | | | | |  | | |  | | | |  | |  |
| Mardani,D. | Int.J.Prev.Med. 2012; 3(6):420-427 |  | | | | X | | | | |  | | |  | | | |  | |  |
| Mariscalco,G. | Ann.Thorac.Surg. 2012; 93(5):1439-1447 |  | | | |  | | | | |  | | |  | | | |  | | V |
| Mesgerani,M. | J.Postgrad.Med.Inst. 2009; 23(4):296-303 |  | | | | X | | | | | X | | |  | | | |  | |  |
| Mu,D. L. | Crit.Care 2010; 14(6):238- | X | | | |  | | | | |  | | |  | | | |  | |  |
| Norkiene,I. | Biomed.Res.Int. 2013; ID# 23491 |  | | | |  | | | | |  | | |  | | | |  | | V |
| Norkiene,I. | Scand.Cardiovasc.J. 2007; 41(3):180-185 |  | | | | X | | | | |  | | |  | | | |  | |  |
| Osse,R. J. | J.Am.Geriatr.Soc. 2012; 60(4):661-668 |  | | | |  | | | | |  | | | X | | | | Case-control | |  |
| Osterbrink,J. | ScientificWorldJournal 2005; 5 874-883 | X | | | | X | | | | | X | | |  | | | |  | |  |
| Otomo,S. | Interact.Cardiovasc.Thorac.Surg. 2013; 17(5):799-804 | X | | | |  | | | | |  | | |  | | | |  | |  |
| Palanzo,D. A. | Perfusion 1996; 11(6):451-453 |  | | | | X | | | | | X | | |  | | | |  | |  |
| Palmbergen,W. A. | Interact.Cardiovasc.Thorac.Surg. 2012; 15(4):671-677 |  | | | |  | | | | |  | | |  | | | |  | | V |
| Park,J.B. | Korean J Thorac Cardiovasc Surg 2014;47:249-254 |  | | | |  | | | | |  | | |  | | | |  | | V |
| Pesonen,A. | Br.J.Anaesth. 2011; 106(6):873-881 | X | | | |  | | | | |  | | |  | | | |  | |  |
| Plaschke,K. | Intensive Care Med. 2010; 36(12):2081-2089 |  | | | | X | | | | | X | | |  | | | |  | |  |
| Prakanrattana,U. | Anaesth.Intensive Care 2007; 35(5):714-719 |  | | | |  | | | | |  | | |  | | | |  | | V |
| Puehler,T. | Eur.J.Cardiothorac.Surg. 2011; 39(4):459-464 | X | | | | X | | | | | X | | |  | | | |  | |  |
| Redelmeier,D. A. | CMAJ 2008; 179(7):645-652 | X | | | | X | | | | |  | | |  | | | |  | |  |
| Redmond,J. M. | Ann.Thorac.Surg. 1996; 61(1):42-47 |  | | | | X | | | | | X | | |  | | | |  | |  |
| Roggenbach,J. | Critical Care 2014, 18:477 |  | | | |  | | | | |  | | |  | | | |  | | V |
| Ronald,A. | Interact.Cardiovasc.Thorac.Surg. 2006; 5(3):207-216 |  | | | |  | | | | |  | | | X | | | | Best evidence review | |  |
| SUBTOTAL |  | 12 | | | | 13 | | | | | 8 | | | 2 | | | |  | | 7 |
| **ADDITIONAL FILE 2: *(Continued)* List of articles screened in full-text review and the rationale for inclusion or exclusion** | | | | | | | | | | | | | | | | | | | | |
| **Author** | **Reference** | **Exclusion reason(s)** | | | | | | | | | | | | | | | |  | |  |
|  |  | **Study population** | | | | **Delirium (Assessment)** | | | | | **Outcome**  **(incl. analysis)** | | | **Other** | | | | **Remarks** | | **Inclusion** |
| Royse,C.F. | Anaesthesia 2011; 66(6):455-464 |  | | | | X | | | | |  | | |  | | | |  | |  |
| Rudolph,J. L. | J.Am.Geriatr.Soc. 2005; 53(3):462-466 |  | | | |  | | | | |  | | |  | | | |  | | V |
| Rudolph,J. L. (A) | Perfusion 2009; 24(6):409-415 |  | | | |  | | | | |  | | |  | | | |  | | V |
| Rudolph,J. L. | J.Am.Geriatr.Soc. 2006; 54(6):937-941 |  | | | |  | | | | |  | | |  | | | |  | | V |
| Rudolph,J. L. (B) | Circulation 2009; 119(2):229-236 | X | | | |  | | | | |  | | |  | | | |  | |  |
| Rudolph,J. L. | J.Gerontol.A Biol.Sci.Med.Sci. 2008; 63(2):184-189 |  | | | |  | | | | |  | | | X | | | | Matched control | |  |
| Saczynski,J. S. | N.Engl.J.Med. 2012; 367(1):30-39 |  | | | | X | | | | | X | | |  | | | |  | |  |
| Santana Santos,F. | Int.Psychogeriatr. 2004; 16(2):175-193 |  | | | |  | | | | |  | | |  | | | |  | | V |
| Sauer,A. C. | Anesth. Analg. (accepted) 2014 |  | | | |  | | | | |  | | |  | | | |  | | V |
| Schmitz,C. | J.Thorac.Cardiovasc.Surg. 2003; 126(6):1829-1838 |  | | | | X | | | | |  | | |  | | | |  | |  |
| Schoen,J. | Crit.Care 2011; 15(5):218 |  | | | |  | | | | |  | | |  | | | |  | | V |
| Schroder-P., S. | Interact.Cardiovasc.Thorac.Surg. 2013; 1-8 | X | | | |  | | | | | X | | |  | | | |  | |  |
| Scott,N. B. | Anesth.Analg. 2001; 93(3):528-535 |  | | | | X | | | | |  | | |  | | | |  | |  |
| Sezer,O. | Klin.Psikofarmakol.Bul. 2004; 14(4):185-190 |  | | | |  | | | | |  | | | X | | | | Turkish | |  |
| Shadvar,K. | J.Cardiovasc.Thorac.Res. 2013; 5(4):157-161 | X | | | |  | | | | | X | | |  | | | |  | |  |
| Shehabi,Y. | Anesthesiology 2009; 111(5):1075-1084 |  | | | |  | | | | |  | | |  | | | |  | | V |
| Shioiri,A. | Am.J.Geriatr.Psychiatry 2010; 18(8):743-753 | X | | | |  | | | | |  | | |  | | | |  | |  |
| Siepe,M. | Eur.J.Cardiothorac.Surg. 2011; 40(1):200-207 |  | | | | X | | | | |  | | |  | | | |  | |  |
| Smulter,N. | Interact.Cardiovasc.Thorac.Surg. 2013; 17(5):790-796 |  | | | |  | | | | |  | | |  | | | |  | | V |
| Stransky,M. | J.Cardiothorac.Vasc.Anesth. 2011; 25(6):968-974 | X | | | |  | | | | |  | | |  | | | |  | |  |
| Suehiro,K. | J.Cardiothorac.Vasc.Anesth. 2014; 28(3): 528–533 | X | | | | X | | | | |  | | |  | | | |  | |  |
| Tagarakis,G. | Clin.Res.Cardiol. 2007; 96(9):600-603 | X | | | | X | | | | | X | | |  | | | |  | |  |
| Tagarakis,G. I. | Am.J.Alzheimers Dis.Other Demen. 2007; 22(3):223-228 | X | | | | X | | | | | X | | |  | | | |  | |  |
| Taipale,P. G. | Int.J.Nurs.Stud. 2012; 49(9):1064-1073 |  | | | |  | | | | |  | | |  | | | |  | | V |
| Tan,M. C. | Am.J.Geriatr.Psychiatry 2008; 16(7):575-583 |  | | | |  | | | | | X | | |  | | | |  | |  |
| Tully,P. J. | Aust.N.Z.J.Psychiatry 2010; 44(11):1005-1011 |  | | | |  | | | | |  | | |  | | | |  | | V |
| van der Mast,R. C. | J.Neuropsychiatry Clin.Neurosci. 2000; 12(1):57-63 |  | | | |  | | | | | X | | |  | | | |  | |  |
| van der Mast,R. C. | J.Psychosom.Res. 1999; 46(5):479-483 |  | | | |  | | | | |  | | |  | | | |  | | V |
| Veliz-Reissmuller,G. | Aging Clin.Exp.Res. 2007; 19(3):172-177 |  | | | |  | | | | |  | | |  | | | |  | | V |
| SUBTOTAL |  | 8 | | | | 8 | | | | | 6 | | | 2 | | | |  | | 12 |
| **ADDITIONAL FILE 2: *(Continued)* List of articles screened in full-text review and the rationale for inclusion or exclusion** | | | | | | | | | | | | | | | | | | | | |
| **Author** | **Reference** | **Exclusion reason(s)** | | | | | | | | | | | | | | | |  | |  |
|  |  | **Study population** | | | | **Delirium (Assessment)** | | | | | **Outcome**  **(incl. analysis)** | | | **Other** | | | | **Remarks** | | **Inclusion** |
| Walzer,T. | J.Neurol.Neurosurg.Psychiatry. 1997; 62(6):644-648 |  | | | | X | | | | |  | | |  | | | |  | |  |
| Wanat,M. | MDCVJ 2014 (2): 111-117 |  | | | |  | | | | | X | | |  | | | |  | |  |
| Whitlock,E.L. | Anesth.Analg. 2014; jan-09 | X | | | |  | | | | |  | | |  | | | |  | |  |
| Yamada,T. | J.Anesth. 2003; 17(3):171-176 |  | | | | X | | | | | X | | |  | | | |  | |  |
| Yildizeli,B. | Ann.Thorac.Surg. 2005; 79(3):1004-1009 | X | | | | X | | | | |  | | |  | | | |  | |  |
| Yoon,B. W. | Stroke 2001; 32(1):94-99 |  | | | | X | | | | | X | | |  | | | |  | |  |
| SUBTOTAL |  | 2 | | | | 4 | | | | | 3 | | | - | | | |  | | - |
|  | | 13 | | | | 12 | | | | | 11 | | |  | | | |  | |  |
|  |  | 9 | | | | | | | | |  | | | | | | |  | |  |
|  |  | 12 | | | | |  | | | | |  | | | | | |  | |  |
|  |  |  | | | | | 18 | | | | |  | | | | | |  | |  |
|  |  | (6) | | | |  | | | | | (6) | | |  | | | | Study population & Outcome | |  |
| **TOTAL** | | **40** | | | | **51** | | | | | **44** | | | **8** | | | |  | | **34** |
