# Supplementary material for: Risk factors for delirium after on-pump cardiac surgery: a systematic review
Source: Crit Care. 2015 Sep 23;19(1):346. doi: 10.1186/s13054-015-1060-0 (PMC4579578; doi:10.1186/s13054-015-1060-0)
Supplement: Additional file 4: — Summary of study characteristics. (DOC 86 kb) [file 13054_2015_1060_MOESM4_ESM.doc]

**ADDITIONAL FILE 3**

Risk factors for delirium after on-pump cardiac surgery: a systematic review

A.N.C. Gosselt, MD., A.J.C. Slooter, MD., PhD., P.R.Q. Boere, MD., I.J. Zaal, MD., PhD.

| **ADDITIONAL FILE 3: Summary of study characteristics** | | | | | | | | | | | | | | |
| --- | --- | --- | --- | --- | --- | --- | --- | --- | --- | --- | --- | --- | --- | --- |
| **Number** | **Author - Year** | **Design** | **Number of delirious / total (%)** | **Type surgery** | **Elective (1) / Urgent (2) / Emergency (3)** | **Definitely with CPB** | **Age specification (years)** | **Delirium assessment tool** | **Assessors** | **Postoperative day assessment** | **Statistical Model** | **Remarks** | | **Quality** |
| 1 | Afonso – 2010 | Cohort | 38/112 (33.9) | Cardiac +/- TA | ? | ? | ≥18 | CAM-ICU | (ICU) Nurse | ICU | LRM | 18% other cardiac | | ++ |
| 2 | Arenson – 2013 | B/A | 148/1010 (14.7) | Cardiac | 1/3 | ? | - | CAM(-ICU) | Standard practice | 1-10 | LRM | 12.2% other cardiac | | - |
| 3 | Burkhart – 2010 | Cohort | 35/113 (30.9) | Cardiac | 1 | + | ≥65 | CAM | Researcher | 1-6 | LRM | Cohort of RCT (#4) | | ++ |
| 4 | Chang – 2008 | Cohort | 120/288 (41.7) | Cardiac +/- TA | 1-3 | + | - | DSM-IV | Psychiatrist | 1-6 | LRM | 11.5% TA patients | | - |
| 5 | Detroyer – 2008 | Cohort | 27/104 (26.0) | Cardiac | 1 | ? | ≥60 | CAM(-ICU) | Researcher | 1/3/7 | LRM | 7.7% other cardiac | | ++ |
| 6 | Eizadi-Mood – 2014 | Cohort | 144/325 (44.3) | CABG +/- valve | 1 | + | ≥18 | ICDSC | Physician | ICU | LRM |  | | + |
| 7 | Gamberini – 2009 | RCT | 35/113 (30.9) | Cardiac | 1 | + | ≥65 | CAM | Researcher | 1-6 | χ^2^ |  | | ++ |
| 8 | Hakim – 2012 | RCT (+) | 24/101 (23.8) | Cardiac | ? | + | ≥65 | ICDSC | Physician | Dis. | Comp. RM | Subsyndromal delirium patients | | ++ |
| 9 | Hudetz – 2009 | RCT | 10/58 (17.2) | CABG a/o valve | 1 | + | ≥55 | ICDSC | Psychologist | 1-5 | Fisher, LRM |  | | ++ |
| 10 | Jung – 2014 | Cohort | 24/133 (18.0) | CABG a/o valve | 1 | ? | ≥18 | CAM(-ICU) | (ICU) Nurse | 1-Dis | RM |  | | ++ |
| 11 | Katznelson – 2009 | Cohort | 122/1059 (11.5) | Cardiac | 1 | + | - | CAM-ICU | (Trained) Nurse | ICU | LRM |  | | ++ |
| 12 | Kazmierski – 2014 | Cohort | 41/113 (36.3) | CABG | 1 | + | - | CAM-ICU | Psychiatrist | 1-5 | LRM | Studies use same cohort | | + |
| 13 | Kazmierski – 2014 | Cohort | 41/113 (36.3) | CABG | 1 | + | - | CAM-ICU | Psychiatrist | 1-5 | LRM | Studies use same cohort | | ++ |
| 14 | Kazmierski – 2013^a^ | Cohort | 41/113 (36.3) | CABG | 1 | + | - | CAM-ICU | Psychiatrist | 1-5 | LRM | Studies use same cohort | | ++ |
| 15 | Kazmierski – 2010 | Cohort | 92/563 (16.3) | Cardiac | 1 | + | ≥18 | DSM-IV | Psychiatrist | 2-6 | LRM | 16 other cardiac | | + |
| 16 | Maldonado – 2009 | RCT(+) | 37/118 (31.4) | Cardiac +/- TA | 1 | + | ≥18 | DSM-IV | Psychiatrist | 1-3 | χ^2^, LRM | 21 TA patients | | +/- |
| 17 | Mariscalco – 2012 | Cohort | 117/4079 (2.9) | CABG +/- valve | 1-3 | + | - | CAM-ICU | Nurse / Physician | ICU | Hierarc.LRM |  | | ++ |
| 18 | Norkiene – 2013 | Cohort | 12/87 (13.3) | Cardiac | 1 | + | - | ICDSC | ? | ICU | LRM |  | | - |
| 19 | Palmbergen – 2012 | B/A | 61/642 (9.5) | CABG +/- other | 1 | + | ≥18 | DOS | Nurse / Physician | ? | LRM |  | | - |
| 20 | Park – 2014 | RCT | 23/142 (16.2) | Valve +/- CABG | 1 | + | ≥17 | CAM-ICU | Researcher | 1-3 | χ^2^ |  | | + |
| 21 | Prakanrattana – 2007 | RCT(+) | 27/126 (21.4) | Cardiac | 1 | + | ≥40 | CAM-ICU | Physician | 1-3(+) | χ^2^, LRM |  | ++/+ | |
| 22 | Roggenbach – 2014 | Cohort | 44/92 (47.8) | CABG a/o valve | 1 | + | >18 | CAM-ICU | Researcher | 1-4 | LRM |  | | ++ |
| **Number** | **Author - Year** | **Design** | **Number of delirious / total (%)** | **Type surgery** | **Elective (1) / Urgent (2) / Emergency (3)** | **Definitely with CPB** | **Age specification (years)** | **Delirium assessment tool** | **Assessors** | **Postoperative day assessment** | **Statistical Model** | **Remarks** | | **Quality** |
| 23 | Rudolph – 2005 | Cohort | 15/36 (41.7) | CABG | 1 | + | * | CAM | Researcher | 2/5 | CRM |  | | + |
| 24 | Rudolph – 2006 | Cohort | 40/80 (50.0) | CABG +/- valve | ? | ? | ≥60 | CAM(-ICU) | Researcher | 2-5 | Poisson |  | | + |
| 25 | Rudolph – 2009 | Cohort | 33/68 (48.5) | CABG. | ? | + | * | CAM | Physician | 2/5 | LRM |  | | + |
| 26 | Santana-Santos - 2004 | Cohort | 75/220 (33.6) | CABG | 1 | + | ≥60 | DSM-IV^b^ | Researcher | 1-5 | LRM |  | | + |
| 27 | Sauer – 2014 | RCT (+) | 107/737 (14.5) | Cardiac | 1 | + | ≥18 | CAM-(ICU) | Researcher | 1-4 | χ^2^, LRM |  | | ++ |
| 28 | Schoen – 2011 | Cohort | 62/231 (26.8) | Cardiac | 1/2 | + | ≥18 | CAM-ICU | Researcher | 0-3 | LRM. | 17% other surgery | | ++ |
| 29 | Shehabi – 2009 | RCT | 35/299 (11.7) | Valve +/- CABG | 1/2 | + | ≥60 | CAM-ICU^b^ | Researcher | 1-5 | CRM |  | | + |
| 30 | Smulter – 2013 | Cohort | 78/142 (54.9) | CABG +/- other | 1 | + | ≥70 | DSM-IV | Researcher | 1/4 | LRM | 5 TA patients | | + |
| 31 | Taipale-2012 | Cohort | 27/122 (22.1) | CABG or valve | 1/2 | + | - | CAM-ICU | Researcher | ICU | LRM | Two delirium definitions | | ++ |
| 32 | Tully – 2010 | Cohort | 49/158 (31.0) | CABG a/o valve | 1 | + | >18 | DSM-IV | Researcher | ? | LRM |  | | + |
| 33 | Van de Mast – 1999 | Cohort | 40/296 (13.5) | Cardiac | 1 | ? | ≥21 | DSMIII-r | Psychiatrist | 2-5 | LRM |  | | + |
| 34 | Veliz-Reissmuller - 2007 | Cohort | 25/107 (23.4) | CABG a/o valve | 1 | + | ≥60 | CAM | Researcher | 2-Dis. | LRM |  | | + |
| ? =unknown, ++=high quality, +=acceptable quality, - = low quality. ^a^ = use same study cohort, ^b^ in combination with Chart review, ^c^ in combination with haloperidol administration. B/A=Before after intervention study, CABG = Coronary Artery Bypass Graft, CAM-ICU=Confusion Assessment Method for the ICU, Comp.RM = competing-risk regression analysis, CRM = Cox regression model, CPB = Cardiopulmonary bypass, Dis.=discharge, DRS = Delirium Rating Scale, DSM=Diagnostic Statistic Manual, FU=Follow-up, ICDSC=Intensive Care Delirium Screening Checklist, ICU=Intensive Care Unit, LRM = Logistic Regression Model, MWU = Mann Whitney U-test, RCT=randomized controlled trial, TA = thoracic aortic surgery, χ^2^ = Pearson’s Chi Square test | | | | | | | | | | | | | | |
