# Supplementary material for: Risk factors for delirium after on-pump cardiac surgery: a systematic review
Source: Crit Care. 2015 Sep 23;19(1):346. doi: 10.1186/s13054-015-1060-0 (PMC4579578; doi:10.1186/s13054-015-1060-0)
Supplement: Additional file 5: — Description of the multivariable statistical model used in included cohort studies. (DOC 120 kb) [file 13054_2015_1060_MOESM5_ESM.doc]

**ADDITIONAL FILE 4**

Risk factors for delirium after on-pump cardiac surgery: a systematic review

A.N.C. Gosselt, MD., A.J.C. Slooter, MD., PhD., P.R.Q. Boere, MD., I.J. Zaal, MD., PhD.

| **ADDITIONAL FILE 4. Description of the statistical model of included cohort studies.** | | | | | | | | |
| --- | --- | --- | --- | --- | --- | --- | --- | --- |
| **Number** | **First Author** | **Year** | | **Study Design** | **Statistical Model** | **Building** | **Multivariable Model** | **Quality** |
| 1 | Afonso | | 2010 | Cohort | Logistic RM  Stepwise | Included | Age, procedure-time | ++ |
|  |  |  |  |  |  | Removed MV | Not described in manuscript |  |
|  |  |  |  |  |  | Removed^b^ | Race, BMI, gender, nicotine / alcohol use, CHF, COPD, DM, ACE-inhibitor, LVEF, CCI, anesthesia time, CPB-time, ACC-time |  |
| 2 | Arenson | | 2013 | B/A | Logistic RM  Stepwise (FW) | Included | Age, benzodiazepine, type of surgery. postop.: stroke or TIA, MV-time, RBC, renal insufficiency | - |
|  |  |  |  |  |  | Removed MV | COPD. Preop.: renal insufficiency, creatinine, albumin, Hb, anemia, surgery urgency, DHCA, redo-sternotomy, CPB-time. Intraop.: desflurane, glycopyrrolate, midazolam, neostigmine, Hb. Postop*.:* MV-time, reoperation, Inotrope/vasopressor/IABP>24 h, cardiac arrhythmia, dialysis, infection, sepsis, sodium, creatinine, RBC, FFP, platelet, cryoprecipitate |  |
|  |  |  |  |  |  | Removed^b^ | Intensive care unit environment. |  |
| 3 | Burkhart | | 2010 | Cohort | Logistic RM  Stepwise (Forward and Backward) | Included | CRP max. Intraop fentanyl, MV-duration | ++ |
|  |  |  |  |  |  | Removed MV | EuroSCORE. Preop.: statins, leucocytes, type of surgery, CPB-time, RBC transfusion. Postop.: treated nausea or vomiting / opiate usage. |  |
|  |  |  |  |  |  | Removed | (Age, concomitant disease, ASA)^d^, Years of education^E^ |  |
| 4 | Chang | | 2008 | Cohort | Logistic RM  Stepwise | Included | Postop.: Albumin, Hct, cardiogenic shock, acute infection | - |
|  |  |  |  |  |  | Removed MV | Age, education, marital status, depression, DM, stroke, renal disease, LVEF, AF, Emergency surgery, cardiogenic shock, type of surgery, duration of circulatory arrest. Intraop.: RBC transfusion, temp. Postop.: LVEF, AF, RBC transfusion, blood loss, reoperation, dehydration, creat, bilirubin, PaCO2, anticholinergic drug. |  |
|  |  |  |  |  |  | Removed^b^ | Gender, BMI, living situation, ethnic group, nicotine / alcohol / benzodiazepine use, HT, PVD, COPD, Comatose, CPB-time, Ischemic time, anesthesia. Postop.: Hypoxemia, sodium, inotropic/steroid/hypnotic/analgesic drug use. |  |
| 5 | Detroyer | | 2008 | Cohort | Logistic RM | Included^f^ | Lowest body temp, MV-time | ++ |
|  |  |  |  |  |  | Removed MV | Living situation, CPB-time, nicotine / alcohol use, serum glucose, Hb, APACHE II, State Anxiety (STAI), depressive symptoms (HADS) |  |
|  |  |  |  |  |  | Removed^b^ | Age, gender, education, type of surgery, DM, psychiatric history, MMSE score, ADL-score. Intraop: lowest / highest (S/D)BP, SatO2, highest temp |  |
| 6 | Eizadi-Mood | | 2014 | Cohort | Logistic RM | Included | Opium addiction, Preop: leucocytes, triglycerides, urea. Postop: creat, fasting BG, urea, dyslipidemia | + |
|  |  |  |  |  |  | Removed MV | Not described in manuscript |  |
|  |  |  |  |  |  | Removed | Age, BMI, education, social status, nicotine use, HT, Angina, previous MI, DM, previous stroke, COPD, depression, constipation. Procedure time, CPB-time, ventilator time, LVEF, reoperation, morphine / pethidine dosage first 24hrs. Postop.: temperature, leucocytes, pH, PaCO2, HCO3, PaO2, MAP, HR, LVEF, urinary output. |  |
| ++ = high quality, + = acceptable quality, - = low quality. ^b^ p-value univariate analysis before stepwise/block regression analysis, ^d^ eliminated to prevent collinearity, only Euroscore included, ^E^ many missing values, ^f^ variables included LR model, only mechanical ventilation time and body temperature significant. ACC = aortic cross-clamping, ACE ° angiotensin converting enzyme, ADL = Activity Daily Living, AF: Atrial Fibrillation, APACHEII = Acute Physiology and Chronic Health Evaluation, ASA = American Society of Anesthesiologists Physical Status Classification System., B/A = Before/After, (S/D)BP = (systolic / diastolic) arterial bloodpressure, BG ° bloodglucose, BMI = Body mass index, CCI = Charlson Comorbidity Index , (C)HF = (congestive) heart failure, COPD = chronic obstructive pulmonary disease, CPB = cardiopulmonary bypass, CRP/C/reactive protein, DHCA = Deep hypothermic circulatory arrest, DM = Diabetes Mellitus, (LV)EF: (Left Ventricle) Ejection Fraction, FFP = fresh frozen plasma, HADS = Hospital Anxiety and Depression Scale, Hb = hemoglobin, Hct: hematocrit, HR = heart rate, HT = Hypertension, IABP = Intra-Aortic balloon pump, Intraop. = intraoperative, L = Leukocytes, MAP: mean arterial pressure , MMSE = Mini Mental State Examination, MV = Mechanical ventilation, Preop. = preoperative, Postop. = postoperative, PVD = peripheral vascular disease, RBC = red blood cell concentrate, RM = Regression Method, STAI = State-Trait Anxiety Inventory, Temp. = temperature, TIA = transient ischemic attack. | | | | | | | | |
| **ADDITIONAL FILE 4. *(continued)* Description of the statistical model of included cohort studies.** | | | | | | | | |
| **Number** | **First Author** | **Year** | | **Study Design** | **Statistical Model** | **Building** | **Multivariable Model** | **Quality** |
| 7 | Hakim | | 2012 | RCT-cohort | Competing Risk RM | Included | Treatment with Risperidone at time of subsyndromal delirium, Rudolph Risk Score *(prior stroke/TIA, GDS, MMSE, albumin)* | ++ |
|  |  |  |  |  |  | Removed | Age, gender, marital status, education, nicotine / alcohol use, previous MI, past cardiac surgery, NYHA, LVEF, AF, anemia. type of surgery, CPB-time, ACC-time, hypothermia on CPB. Postop.: LVEF, PaO2, Hct, albumin, MV-time, Neostigmine/glycopyrrolate used |  |
| 8 | Jung | | 2014 | Cohort | Logistic RM | Included | Frailty (several different assessment tools), EuroSCORE II |  |
| 9 | Katznelson | | 2009 | Cohort | Logistic RM  Multistep | Included | Age, renal dysfunction, depression, statins, type of surgery, IABP support, RBC transfusion | ++ |
|  |  |  |  |  |  | Removed MV | Gender, HT, PVD, NYHA, DM, stroke/TIA. Preop.: anaemie. CPB-time. Intraop.: Hb / serum glucose |  |
|  |  |  |  |  |  | Removed^b^ | CPB Sodium |  |
| 10 | Kazmierski (A) (Dement. Geriatr. Cogn. Disord.) | | 2014 | Cohort | Logistic RM  Stepwise (FW) | Included | MCI, Dementia, Intraop.: Hb. Postop.: AF / PaO2 | + |
|  |  |  |  |  |  | Removed MV | Age, Depression, MoCA score. Preop.: homocysteine / urea / creatinine / anemia / cortisol. Perfusion time, Midazolam dosage, Postop.: cortisol / TNF- *α* / IL-2 / PaCO2 |  |
|  |  |  |  |  |  | Removed^b^ | Gender, PVD, CVD, HT, DM, NYHA, CCS, Procedure time, ACC-time. Preop.: AF / Cobalamin. Intraop: hemofiltration / ACT. Postop.: temp. |  |
| 11 | Kazmierski (B)  (int. psych. Ger.) | | 2014 | Cohort | Logistic RM  Stepwise (FW) | Included | Depression, TMT-B score, midazolam dosage, reperfusion time Postop.: IL-2 / TNF-*α* / AF. | ++ |
|  |  |  |  |  |  | Removed MV | Age, Education, MoCA score. Preop.: creatinine / anemia / urea. perfusion time, |  |
|  |  |  |  |  |  | Removed^b^ | Gender, PVD, CVD, HT, DM, NYHA, CCS, Procedure time, ACC-time. Preop.: AF. Intraop: hemofiltration / ACT. Postop.: temp / LVEF. |  |
| 12 | Kazmierski  (Crit care) | | 2013 | Cohort | Logistic RM  Stepwise Forward | Included | TMT-B score, depression. Preop.: cortisol / creatinine. Postop.: IL-2. Intraop.: midazolam doze | ++ |
|  |  |  |  |  |  | Removed MV | Age, MoCA, preop.: ureum / Hb, postop.: cortisol / AF. |  |
|  |  |  |  |  |  | Removed^b^ | Gender, Education, CVD, HT, DM, NYHA class, CCS, procedure time, ACC-time. Intraop.: lowest Hb / hypercapnia / hypoxia |  |
| 13 | Kazmierski | | 2010 | Cohort | Logistic RM  Stepwise Backward | Included | Age, MMSE, Depression, Anemia, AF, Prolonged intubation, postop. hypoxia | + |
|  |  |  |  |  |  | Removed MV | CVD, PVD, DM, CCS, NYHA, Type of surgery. Preop.: ureum. Intraop.: hemofiltration / hypercapnia / hypoxia / temp. / periods of hypotonia. Perfusion time, reperfusion time. Postop.: cardiac arrhythmia / RBC / FFP / pCO2 / pO2 / serum urea. MV-time, reoperation. |  |
|  |  |  |  |  |  | Removed^b^ | Gender, history of psychiatic illness, alcohol / nicotine use, HT, prior MI. Preop.: creatinine / pacemaker / arrhythmia. Procedure time, ACC-time, circulatory support, resuscitation. Intraop.: ACT. Postop.: MI, temp., sodium, serum glucose, serum urea |  |
| 14 | Maldonado | | 2009 | RCT - Cohort | Logistic RM (Block) | Included | Age, gender, ASA, postop.: Sedative treatment (Dexmedetomide/Propofol/Midazolam) | + |
|  |  |  |  |  |  | Removed^b^ | History of psychiatic illness, MMSE, TMT, CPB-time, ACC-time, anesthesia-time, procedure-time. Intraop.: fentanyl. |  |
| ++ = high quality, + = acceptable quality. ^b^ p-value univariate analysis before stepwise/block regression analysis. ACC = aortic cross-clamping, ACE ° angiotensin converting enzyme, ACT: Activated Clotting Time, AF: Atrial Fibrillation, ASA = American Society of Anesthesiologists Physical Status Classification System, CCS: Canadian Cardiovascular Society degree, COPD = chronic obstructive pulmonary disease, CPB = cardiopulmonary bypass, CVD = Cerebrovascular disease, DM = Diabetes Mellitus, (LV)EF: (Left Ventricle) Ejection Fraction, FFP = fresh frozen plasma, GDS = General Depression Scale, Hb = hemoglobin, Hct: hematocrit, HT = Hypertension, IL-2: Interleukin 2, Intraop. = intraoperative, MCI: Mild Cognitive Impairment, MI = Myocard infarction, MMSE = Mini Mental State Examination, MoCA: Montreal Cognitive Assessment, MV = Mechanical ventilation, NYHA = New York Heart Association, Preop. = preoperative, Postop. = postoperative, PVD = peripheral vascular disease, RCT= randomized controlled trial, RM = Regression Method, Temp. = temperature, TIA = transient ischemic attack, TMT-(A/B): Trail Making Test Part (A/B), TNF-α: Tumor Necrosis Factor-α. | | | | | | | | |
| **ADDITIONAL FILE 4. *(continued)* Description of the statistical model of included cohort studies.** | | | | | | | | |
| **Number** | **First Author** | **Year** | | **Study Design** | **Statistical Model** | **Building** | **Multivariable Model** | **Quality** |
| 15 | Mariscalco | | 2012 | Cohort | Logistic RM  Stepwise (Forward and Backward) | Included | Age, LVEF classes, HT, COPD, history of stroke, type of operation. Post-op: AF, AKI. | ++ |
|  |  |  |  |  |  | Removed MV | Body surface area, emergency surgery, cardiogenic shock, dyslipidemia, DM, PVD, renal function., b-blocker therapy, calcium antagonists, statins, CPB time, ACC-time. Postop. IABP / inotropic support / RBC / FFP / platelets. |  |
|  |  |  |  |  |  | Removed^b^ | Gender, previous MI, left main stem stenosis, ACE inhibitors. Postop.: acute MI. |  |
| 16 | Norkiene | | 2013 | Cohort | Logistic RM | Included | MV-time, duration ICU-stay | - |
|  |  |  |  |  |  | Removed MV | Age, anesthesia time, procedure time, lactate level, reinstitution of CPB |  |
|  |  |  |  |  |  | Removed^b^ | BMI, nicotine / alcohol abuse, sleep disturbances, MMSE (score), EuroSCORE, STS risk score, DM, CHF, HT, AF, ICA stenosis, COPD, visual or hearing impairment, history of falls, CPB-time, ACC-time, reinstitution of CPB. Intraop.: temp. / MAP / inotropic use. MV-time. Postop.: fluid balance, drainage, VAS, Hb, Hct, lactate, RBC / FFP / Inotropic use / re-sternotomy / rhythm disorders / low cardiac output / CNS events |  |
| 17 | Palmbergen | | 2012 | B/A | Logistic RM | Included^g^ | Age, gender, preop. delirium risk score, type of surgery, HBCS | - |
| 18 | Prakanrattana | | 2007 | RCT - Cohort | Logistic RM | Included | Age, NYHA, postop.: Time to follow commands, respiratory failure, renal failure | + |
|  |  |  |  |  |  | Removed^b^ | Gender, Weight, HT, DM, stroke, renal function, AF, Type of surgery, Anesthesia-time, CPB-time, ACC-time, postop.: time to open eyes, renal failure, AF, cardiovascular instability |  |
| 19 | Roggenbach | | 2014 | Cohort | Logistic LRM | Included | AHI, tabaco use, age, preop.: albumin, Hb. Intraop.: RBC. | ++ |
|  |  |  |  |  |  | Removed^b^ | Gender, BMI, alcohol / longterm steroid / benzodiazepine use, DM, HT, COPD, renal insufficiency. Preop.: LVEF, stroke, AF, urea. type of surgery, CPB-time, MV-time, ICU-time. |  |
| 20 | Rudolph | | 2005 | Cohort | Cox regression | Included | Age, CCI, baseline MMSE, Atherosclerosis score. | + |
|  |  |  |  |  |  | Removed | HT, hyperlipidemia, DM, BMI, nicotine use |  |
| 21 | Rudolph | | 2006 | Cohort | Poisson RM |  | Composite memory and executive functioning adjusted for age, gender, education, CCI.  (Separately without MMSE adjustment) | + |
| 22 | Rudolph | | 2009 | Cohort | Logistic RM | Included | Age, CCI, MMSE, carotid stenosis, ascending aortic plaque, micro-embolic load | + |
|  |  |  |  |  |  | Removed | Gender, BMI, LVEF. Preop.: Hct, Procedure time, CPB-time. Intraop.: Cell saver use, Hct, temp. ACC-time, number of bypasses, ICU admission Hct |  |
| 23 | Santana-Santos | | 2004 | Cohort | Logistic RM  Stepwise | Included^h^ | Age, urea, cardiothoracic index, HT, nicotine use, RBC | + |
|  |  |  |  |  |  | Removed MV | ASA, renal dysfunction, diuretics, ACE-inhibitors. Preop.: creatinine. Intraop.: benzodiazepine premedication, Swan-Ganz, blood loss. |  |
|  |  |  |  |  |  | Removed^b^ | Gender, education, socioeconomic status, DM, COPD, obesitas, dyslipidemia, previous MI, heart failure, LVEF, calcium antagonists, beta blockers, nitrates, ACE-inhibitors, H2 blockers, GDS. Preop.: Hb / Hct / serum glucose / sodium / potassium. CPB-time, ACC-time, CPB-flow, number of grafts, Anesthesia time. Intraop.: RBC / MAP / electrolyte imbalance / acid imbalance. Prolonged CPB, blood loss, IABP, temp, diuresis, fluid balance, |  |
| ++ = high quality, + = acceptable quality, - = low quality. ^b^ p-value univariate analysis before stepwise/block regression analysis. ^g^ decided a priori, ^h^ describe several multivariable models; included without postop. measurement, ^I^ not included intra- / postoperative measurements. ACC = aortic cross-clamping, ACE = angiotensin converting enzyme, AF: Atrial Fibrillation, AHI: apnea-hypopnea index, AKI = acute kidney injury, ASA = American Society of Anesthesiologists Physical Status Classification System., B/A = Before/After, BMI = Body mass index, CCI = Charlson Comorbidity Index , (C)HF = (congestive) heart failure, CNS = central nervous system, COPD = chronic obstructive pulmonary disease, CPB = cardiopulmonary bypass, DM = Diabetes Mellitus, (LV)EF: (Left Ventricle) Ejection Fraction, FFP = fresh frozen plasma, Hb = hemoglobin, HBCS = Haga Brain Care Strategy, Hct: hematocrit, HT = Hypertension, ICA: internal carotid artery, ICU = intensive care unit, Intraop. = intraoperative, MAP: mean arterial pressure, MMSE = Mini Mental State Examination, NYHA = New York Heart Association, Preop. = preoperative, Postop. = postoperative, RBC = red blood cell concentrate, RCT= randomized controlled trial, RM = Regression Method, STS = Society of Thoracic Surgeons, Temp. = temperature, TIA = transient ischemic attack, TMT-(A/B): Trail Making Test Part (A/B). VAS = visual analogue Scale. | | | | | | | | |
| **ADDITIONAL FILE 4. *(continued)* Description of the statistical model of included cohort studies.** | | | | | | | | |
| **Number** | **First Author** | **Year** | | **Study Design** | **Statistical Model** | **Building** | **Multivariable Model** | **Quality** |
| 24 | Sauer | | 2014 | RCT-Cohort | Logistic RM  Stepwise | Included^g^ | Age, gender, history of stroke, type of surgery, intraop. dexamethason bolus. | ++ |
|  |  |  |  |  |  | Removed | Weight, HT, DM, COPD, PVD, recent MI, creatinine, EuroSCORE, LVEF, MV- time, CPB-time, ACC-time, Re-operation |  |
| 25 | Schoen | | 2011 | Cohort | Logistic RM | Included | Age, MMSE, additive EuroScore, neurological/psychiatrical disease. Preop.: Hb / NTproBNP / Baseline cerebral oxygen saturation with supplemental oxygen | ++ |
|  |  |  |  |  |  | Removed^b^ | Gender, BMI, education, LVEF, DM, cerebral vessel stenosis, dementia, type of surgery, preop.: CRP, L, Creatinine. (CPB-time, ACC-time, intraop.: min. Hb, transfusion. Postop.: myocardial injury, inflammation, renal function, dialysis, IABP use, reintubation rate, MV-time)^i^ |  |
| 26 | Smulter^£^ | | 2013 | Cohort | Logistic RM  (Backward and Forward) | Included | DM, Preop.: SatO2 / NRS pain. Type of surgery | + |
|  |  |  |  |  |  | Removed MV | Age, gender, weight, gastritis / peptic ulcer. Preop.: SaO2 / T. |  |
|  |  |  |  |  |  | Removed^b^ | Length, BMI, MMSE, GDS-15, living situation, nicotine use, HT, NYHA, angina, platelet inhibitor, CHF, MI, main stem stenosis, PVD, LVEF, Previous cardiac surgery or PCI, stroke, sleeping disorder, COPD, renal disease, rheumatic-, prostatic-, urogenital-, thyroid-, malignant- or infectious disease.  Preop.: (S/D)BP / temp. / pulse rate / sodium / potassium / creat / L / Hb / T. Procedure time, CPB time, Aortic plaque. Intraop.: lowest (S/D)BP / Arrhythmia / serum glucose / Tranexamic acid / Inotropic usage / RBC / diuresis / Sat O2 / insulin / blood loss / volume load. Postop.: lowest (S/D)BP / pulse rate / blood loss / RBC / Potassium / Creat / L / Hb / T / temp / Sat O2 / sodium / Troponin-T. MV-time |  |
| 27 | Taipale^¥^ | | 2012 | Cohort | Logistic RM  (Backward) | Included | Age, Midazolam, PVD | ++ |
|  |  |  |  |  |  | Removed MV | Gender, HT, Intraop.: temp. |  |
|  |  |  |  |  |  | Removed^b^ | Gender, nicotine use, HT, AF, CHF, DM, stroke or TIA, type of surgery, pulmonary artery catheter Intraop.: blood loss. Postop.: inotropes, hypotension, AF, low cardiac output, hypoxia or hypercarbia |  |
| 28 | Tully | | 2010 | Cohort | Logistic RM  (Block) | Included | Age, gender, depressive disorder, panic disorder, generalized anxiety, Type D personality. ACC-time. Preop.: Hb / composite of psychotropic/anticholinergic drug. | + |
|  |  |  |  |  |  | Removed^b/g^ | Aboriginal, COPD, recent MI, hypercholesterolaemia, HT, DM, renal disease, PVD, LVEF, type of surgery |  |
| 29 | Van de Mast | | 1999 | Cohort | Logistic RM  (Backward) | Included | Age, GHQ-score, nifedipine, albumin, Ratio Phe:oLNAA | + |
|  |  |  |  |  |  | Removed MV | MMSE, inclusion as in-patient, DAL-score, |  |
|  |  |  |  |  |  | Removed^b^ | Ratio rT3:T3, thiamine, alcohol usage. Preop.: Sodium, potassium, serum glucose. |  |
| 30 | Veliz-Reissmuller | | 2007 | Cohort | Logistic RM | Included | Age, alcohol, memory complaints, type of surgery, MMSE | + |
|  |  |  |  |  |  | Removed^j^ | DM, HT, CVD, carotid artery stenosis, angina pectoris, previous MI, COPD, hyperlipidemia, nicotine use, BMI, number of drugs, number of bypasses, procedure-time, CPB-time, ACC-time, intubation time, postop.: AF, MI, transfusion, infection, mediastinitis, Stroke/TIA, AKI. |  |
| ++ = high quality, + = acceptable quality. ^b^ p-value univariate analysis before stepwise/block regression analysis.  ACC = aortic cross-clamping, AF: Atrial Fibrillation, (S/D)BP = (systolic / diastolic) arterial bloodpressure, BMI = Body mass index, (C)HF = (congestive) heart failure, COPD = chronic obstructive pulmonary disease, CPB = cardiopulmonary bypass, CRP = C-reactive protein, DAL = Daily Activity List, DM = Diabetes Mellitus, (LV)EF: (Left Ventricle) Ejection Fraction, FFP = fresh frozen plasma, GDS = geriatric derpression scale, GHQ = General Health Questionnaire, Hb = hemoglobin, Hct: hematocrit, HT = Hypertension, IABP = Intra-Aortic balloon pump, Intraop. = intraoperative, L = Leukocytes, MI = myocardial infarction, MMSE = Mini Mental State Examination, MV = Mechanical ventilation, NRS = numeric rating scale, NYHA = New York Heart Association, Preop. = preoperative, Postop. = postoperative, PVD = peripheral vascular disease, Ratio rT3:T3 = inactive reverse T3 hormone: active T3 hormone; Ratio Phe:oLNAA = ratio phenylalanine to the sum of tryptophan, tyrosine, valine, leucine, and isoleucine, RBC = red blood cell concentrate, RCT= randomized controlled trial, RM = Regression Method, SatO2 = Oxygen saturation, T = thrombocytes, Temp. = temperature, TIA = transient ischemic attack. | | | | | | | | |
