# Supplementary material for: Risk factors for delirium after on-pump cardiac surgery: a systematic review
Source: Crit Care. 2015 Sep 23;19(1):346. doi: 10.1186/s13054-015-1060-0 (PMC4579578; doi:10.1186/s13054-015-1060-0)
Supplement: Additional file 6: — Results of the methodological assessment of controlled trials. (DOC 35 kb) [file 13054_2015_1060_MOESM6_ESM.doc]

**ADDITIONAL FILE 5**

Risk factors for delirium after on-pump cardiac surgery: a systematic review

A.N.C. Gosselt, MD., A.J.C. Slooter, MD., PhD., P.R.Q. Boere, MD., I.J. Zaal, MD., PhD.

| **ADDITIONAL FILE 5: Result of methodological quality assessment of controlled trials.** | | | | | | | | | | | | | |
| --- | --- | --- | --- | --- | --- | --- | --- | --- | --- | --- | --- | --- | --- |
| Number |  | | Random allocation | Concealment | Blinding | Successful randomization | Similar treatment groups | Outcomes | % drop tout | ITT analysis | Comparable across sites | Total | Statement  (-/+/++) |
|  | Author / year of publication | | 1.2 | 1.3 | 1.4 | 1.5 | 1.6 | 1.7 | 1.8 | 1.9 | 1.10 |  |  |
| 7 | Gamberini – 2009 | | 1 | 1 | 1 | 1 | 1 | 0^a^ | 1 (6%) | 1 | n.a. | 8/9 | ++ |
| 8 | Hakim – 2012 | | 1 | 1 | 1 | 1 | 1 | 1 | 1 (11%) | 1 | n.a. | 9/9 | ++ |
| 9 | Hudetz – 2009 | | 1 | 1 | 1 | 1 | 1 | 1 | 1 (8%) | 0 | n.a. | 8/9 | ++ |
| 13 | Maldonado - 2009 | | 1 | ? | 0 | 1 | 0 | 1 | 0 (24%) | 1 | n.a. | 5/9 | - |
| - More postoperative morphine usage in Midazolam compared to Dexmedetomidine group. | | | | | | | | | | | | | |
| 20 | | Park – 2014 | 1 | ? | 0 | 1 | 1 | 1 | 1 (0%) | n.a. | n.a. | 7/9 | + |
| 21 | | Prakanrattana – 2007 | 1 | 1 | 1 | 1 | 1 | 1 | 1 (0%) | n.a. | n.a. | 9/9 | ++ |
| 27 | | Sauer – 2014 | 1 | 1 | 1 | 1 | 1 | 1 | 1 (4%) | 1 | n.a. | 9/9 | ++ |
| 29 | | Shehabi – 2009 | 1 | 1 | 1 | 1 | 0 | 1 | 1 (2%) | 1 | ? | 7/9 | + |
| - More postoperative propofol / midazolam in morphine group. | | | | | | | | | | | | | |
| ++ = high quality; + = average quality; - = low quality; ICU = intensive care unit; SIGN = Scottish Intercollegiate Guidelines Network; ITT = Intention to treat | | | | | | | | | | | | | |

^a^ CAM not validated for ICU use.
