# Supplementary material for: Risk factors for delirium after on-pump cardiac surgery: a systematic review
Source: Crit Care. 2015 Sep 23;19(1):346. doi: 10.1186/s13054-015-1060-0 (PMC4579578; doi:10.1186/s13054-015-1060-0)
Supplement: Additional file 7: — Results of the methodological assessment of cohort studies. (DOC 71 kb) [file 13054_2015_1060_MOESM7_ESM.doc]

**ADDITIONAL FILE 6**

Risk factors for delirium after on-pump cardiac surgery: a systematic review

A.N.C. Gosselt, MD., A.J.C. Slooter, MD., PhD., P.R.Q. Boere, MD., I.J. Zaal, MD., PhD.

| **ADDITIONAL FILE 6: Result of methodological quality assessment of Cohort Studies.** | | | | | | | | | | | | | |
| --- | --- | --- | --- | --- | --- | --- | --- | --- | --- | --- | --- | --- | --- |
| Number | |  | | Low risk of selection bias | Low risk of performance bias | Outcome clearly defined | Outcome assessment blind for exposure | Clear measure of exposure | Clear measure of outcome | Exposure level measured >1 | Statistical analysis | Total | Statement |
|  | | Author - Year of publication | | 1.2 | 1.4 | 1.7 | 1.8 | 1.10 | 1.11 | 1.12 | 1.13 |  |  |
| 1 | | Afonso – 2010 | | 1 | 1 | 0^a^ | n.a. | 1 | 1 | n.a. | 1 | 7/8 | ++ |
| 2 | Arenson – 2013 | | | 0^b^ | 0^c^ | 1 | 0^d^ | 1 | 1^+^ | 1 | 0^++^ | 4/8 | - |
|  | | | | ^+^ CAM-(ICU) standard practice; assessor not described.  ^++^ ICU environment not included multivariate model, despite significant differences. | | | | | | | | | |
| 3 | Burkhart – 2010 | | | 1 | 1 | 1 | n.a. | 1 | 0^+^ | n.a. | 1 | 7/8 | ++ |
|  | | | | ^+^ CAM not validated for ICU | | | | | | | | | |
| 4 | Chang – 2008 | | | 1 | 0^c^ | 1 | n.a. | 0^+^ | 0^++^ | n.a. | 0^+++^ | 4/8 | - |
|  | | | | ^+^ Cardiogenic shock / sepsis no valid definition given.  ^++^ Retrospective chart review according to DSM-IV.  ^+++^ Model includes post delirium variables / invalid confidence interval provided | | | | | | | | | |
| 5 | Detroyer – 2008 | | | 1 | 1 | 1 | n.a. | 1 | 1 | 1 | 1 | 8/8 | ++ |
| 6 | Eizadi-Mood – 2014 | | | 1 | 1^c^ | 0^a^ | 1 | 0 | 1 | n.a. | 0^+^ | 5/8 | + |
|  | | | ^+^ many significant variables not included in the model | | | | | | | | | | |
| 8 | Hakim – 2012 | | | 1^+^ | 1 | 1 | 1 | 1 | 1 | n.a. | 1 | 8/8 | ++ |
|  | | | | ^+^ Subsyndromal delirium only. | | | | | | | | | |
| 10 | Jung – 2014 | | | 1 | 1^c^ | 1 | 1 | 1 | 1 | n.a. | 0^+^ | 7/8 | ++ |
|  | | | | ^+^ adjusted for Euroscore II only | | | | | | | | | |
| 11 | Katznelson – 2009 | | | 1 | 1^c^ | 0^a^ | 1 | 1 | 1 | n.a. | 1 | 7/8 | ++ |
| 12 | Kazmierski – 2014 (A)^¥^ | | | 0^b^ | 1 | 1 | 0^h^ | 1 | 1^e^ | 1 | 1 | 6/8 | + |
| 13 | Kazmierski – 2014 (B)^¥^ | | | 0^b^ | 1 | 1 | n.a. | 1 | 1^e^ | 1 | 1 | 7/8 | ++ |
| 14 | Kazmierski – 2013^¥^ | | | 0^b^ | 1 | 1 | n.a. | 1 | 1^e^ | 1 | 1 | 7/8 | ++ |
| 15 | Kazmierski – 2010 | | | 0^b^ | 1 | 0^f^ | 1 | 1 | 1 | ? | 1 | 5/8 | + |
| 16 | Maldonado – 2009^£^ | | | 0^+^ | 1 | 1 | 0 | 1 | 1 | n.a. | 1 | 6/8 | + |
|  | | | | ^+^ 24% dropout. | | | | | | | | | |
| 17 | Mariscalco – 2012 | | | 1 | 1^c^ | 0^a+^ | 1 | 1 | 1 | n.a. | 1 | 7/8 | ++ |
|  | | | | ^+^ Delirium CAM-ICU ≥2 positive. | | | | | | | | | |
| 18 | Norkiene – 2013 | | | 0^+^ | 1 | 0^a++^ | n.a. | 0^+++^ | 1 | n.a | 0> | 4/8 | - |
|  | | | | ^+^ 87/110 Included; 79 patients in results.  ^++^ Excluding ICU stay <24hours.  ^+++^ Several variables not defined.  > Final model only length of stay / mechanical ventilation. | | | | | | | | | |
| ++ = High quality (7-8), + = acceptable quality (5-6), - = low quality (≤ 4), 1=yes, 0=no, ?=can’t say, n.a.= not applicable.  ^a^ ICU assessment only. ^b^ Exclusion died within study period. ^c^ No preoperative delirium screening, however elective surgery only. ^d^ Before / after without blinding. ^e^  CAM-ICU used at the ward. ^¥^ all studies used same cohort. ^£^ multivariate cohort assessment in controlled trial. CAM(-ICU) = Confusion Assessment Method (adjusted for Intensive Care Unit). DSM = Diagnostic and Statistical Manual of Mental Disorders | | | | | | | | | | | | | |
| 19 | Palmbergen – 2012 | | | 0^+^ | 1^c^ | 0^++^ | 0^d^ | 0^+++^ | 0> | n.a. | 1 | 3/8 | - |
|  | | | | ^+^ Full preoperative workup only.  ^++^ Follow-up time not provided.  ^+++^ Preoperative delirium score not validated.  > Probably DOS at ICU stay | | | | | | | | | |
| 21 | Prakanrattana –2007^£^ | | | 1 | 1 | 0^+^ | n.a. | 0^++^ | 1 | n.a. | 1 | 6/8 | + |
|  | | | | ^+^ Follow-up not clearly defined.  ^++^ ‘Time from opening eyes to following commands’ and ‘respiratory failure’ not further defined. | | | | | | | | | |
| 22 | Roggenbach – 2014 | | | 1 | 1^i^ | 1 | 1 | 1 | 1 | n.a. | 1 | 8/8 | ++ |
| 23 | Rudolph –2005 | | | 1 | 1 | 0^f^ | 1 | 1 | 0^g^ | n.a. | 1^+^ | 6/8 | + |
|  | | | | ^+^ Cox Regression postoperative day 2 only. | | | | | | | | | |
| 24 | Rudolph – 2006 | | | 1 | 1 | 0^f^ | ?^h^ | 1 | 1 | n.a. | 1 | 6/8 | + |
| 25 | Rudolph – 2009 | | | 1 | 1 | 0^f^ | ?^h^ | 1 | 0^g^ | n.a. | 1 | 5/8 | + |
| 26 | Santana-Santos – 2004 | | | 1 | 1^c/i^ | 0^f^ | n.a. | 0^+^ | 1 | n.a. | 1^++^ | 6/8 | + |
|  | | | | ^+^ Cardiothoracic index measurement not specified.  ^++^ included the pre- and intraoperative model. | | | | | | | | | |
| 27 | Sauer – 2014^£^ | | | 1 | 1^c^ | 1 | 1 | 1 | 1 | n.a. | 1 | 8/8 | ++ |
| 28 | Schoen – 2011 | | | 0^b^ | 1 | 1 | 1 | 1 | 1 | 1 | 1 | 7/8 | ++ |
| 30 | Smulter – 2013 | | | 0^b^ | 1 | 0^+^ | 1 | 1 | 1 | n.a. | 1 | 6/8 | + |
|  | | | | ^+^ Postoperative day 1 en 4; likely missed delirium day 2/3. | | | | | | | | | |
| 31 | Taipale – 2012 | | | 1 | 1^i^ | 1 | 1 | 1 | 1^e^ | 1^+^ | 1 | 8/8 | ++ |
|  | | | | ^+^ Hypoxia / hypercarbia / low cardiac output measured once. | | | | | | | | | |
| 32 | Tully – 2010 | | | 1 | 1 | 0^+^ | ?^h^ | 1 | 1^++^ | n.a. | 1 | 6/8 | + |
|  | | | | ^+^ Measurement interval not provided. ^++^ Motivated adjustment of criteria. | | | | | | | | | |
| 33 | Van der Mast – 1999 | | | 0^b^ | 1^c/i^ | 0^f^ | 1 | 1 | 1 | n.a. | 1 | 6/8 | + |
| 34 | Veliz-Reissmuller – 2007 | | | 1 | 1 | 0^f^ | 0^+^ | 1 | 0^g^ | n.a. | 1 | 5/8 | + |
|  | | | | ^+^  All assessments done by one person. | | | | | | | | | |
| ++ = High quality (7-8), + = acceptable quality (5-6), - = low quality (≤ 4), 1=yes, 0=no, ?=can’t say, n.a.= not applicable.  ^a^ ICU stay only. ^b^ Exclusion died within study period. ^c^ No preoperative delirium screening, however elective surgery only. ^d^ Before / after without blinding. ^e^  CAM-ICU used at the ward. ^f^ Missed postoperative day 1 assessment. ^g^ CAM possibly still at ICU / ventilated patients. ^h^ Delirium assessment (likely) not blind for psychological tests / embolic load. ^i^ Cognitive impairment excluded. ^¥^ all studies used same cohort. ^£^ multivariate cohort assessment in controlled trial. DOS = Delirium Observation Scale. | | | | | | | | | | | | | |
